# Particle settling in a shear-thinning, viscoelastic fluid in the presence of wall effects

**DOI:** 10.1038/s41598-025-87742-w

**Published:** 2025-02-06

**Authors:** Jodie Whorton, Thomas J. Jones, James K. Russell

**Affiliations:** 1 Lancaster Environment Centre, Lancaster University, Lancaster, LA1 4YQ UK; 2 Earth, Ocean and Atmospheric Sciences, The University of British Columbia, Vancouver, BC V6T 1Z4 Canada

**Keywords:** Fluid dynamics, Rheology, Chemical engineering

## Abstract

The settling of particles in fluids is a widespread phenomenon and commonly involves accounting for the effects of walls. Particle settling and wall effects are well understood for Newtonian fluids but the consequences of non-Newtonian fluid properties on particle settling are less well known. Here, we present the results from a set of experiments quantifying wall effects on particle settling within quiescent shear-thinning and viscoelastic (non-Newtonian) fluids for sphere-to-tube diameter ratios $\lambda \le 0.3$. We find that wall effects on particle settling are reduced in non-Newtonian fluids and settling velocities are poorly predicted by conventional wall-corrected Stokes’ equations. We show that deviations in settling velocity are due to both the shear-thinning and viscoelastic properties of the fluid. Supported by our experimental dataset, we are able to show that calculating the shear-rate based on the particle diameter length-scale corresponds to an apparent viscosity that appropriately accounts for shear-thinning effects. A further correction factor for viscoelastic behaviour based on $\lambda$ and the Weissenberg number, Wi, is applied, and shows good agreement with all experimentally measured velocities. Together, we provide a quantitative method to accurately predict the terminal settling velocity of particles in shear-thinning, viscoelastic fluids up to sphere-to-tube diameter ratios of 0.3.

## Introduction

Particle settling in fluids is one of the longest studied physical processes in theoretical fluid mechanics^1^, followed by studies of the effects of walls on particle settling rates^2^. Settling of particles in fluids has received such a high level of attention because the process operates in so many natural environments^3^, for example, from microscopic red blood cells suspended in plasma^4^, ash dispersal in volcanic plumes^5^, sediment transport in rivers^6^, to rock fragments and crystals in natural magmas^7,8^. Particle settling is also a critical component to numerous industrial applications. These include, for example, the transportation of slurries, mineral processing, drilling, and waste water treatment^9,10^. In 1851, George Gabriel Stokes developed an equation for calculating the terminal velocity of a perfectly spherical particle falling through an unbounded fluid with a constant Newtonian viscosity^1^. A particle reaches its terminal settling velocity when the weight of the particle is balanced with the total buoyancy and resisting fluid force around the particle^11^. The acceleration becomes zero and the particle falls at a constant (i.e., terminal) velocity. The terminal velocity of the particle is set by the density contrast between fluid and particle, particle size, and the viscosity through the fluid. Many situations involve particles settling in fluids where there are physical boundaries (i.e., walls) at short distances relative to the particle diameter. In these situations, the effects of the walls causes significant deviations between the measured settling velocity and the velocity predicted by Stokes, requiring corrections to Stokes’ Law^2^. Experimental studies of the wall effect for spherical particles falling in cylindrical tubes for Newtonian fluids include Bacon^12^, Fayon and Happel^13^, Fidleris and Whitmore^2^, Sutterby^14^, Achenbach^15^, Iwaoka and Ishii^16^, Modi and Akutsu^17^, Lali et al.^18^, Humphrey and Murata^19^, Bougas and Stamatoudis^20^, Uhlherr and Chhabra^21^, Ataide et al.^22^, Kehlenbeck and DiFelice^23^, and Arsenijevic et al.^24^. Together, these experimental studies have shown that the presence of boundary walls produces a retardation effect and reduces the terminal settling velocity of a particle. Furthermore, numerical studies show the wall factor, *f* (the ratio of the bounded settling velocity to the unbounded settling velocity), to be a function of $\lambda$, the sphere-to-tube diameter ratio, in low and high Reynolds number regimes^25^ (see Table 1 for a full list of symbols used in this study). A limited amount of results are available for the intermediate Reynolds number regime^26^, however, here we focus on the low Reynolds number regime due to its widespread applicability to numerous natural and industrial settings.

**Table 1:** Symbols used in this study, ordered alphabetically with Roman letters followed by Greek.

Symbol	Parameter	Units
*A*	Particle cross-sectional area	$\hbox {m}^{2}$
$A_0$	Empirical constant, see Eq. 9	–
*B*	Empirical constant, see Eq. 10	–
*c*	Polymer Cross constant	–
*C*	Empirical constant, see Eq. 11	–
$C_d$	Drag coefficient	–
*d*	Particle diameter	m
*D*	Cylindrical tube diameter	m
*De*	Deborah number	–
*f*	Wall factor	–
$f_0$	Viscoelastic wall correction factor	–
$F_D$	Drag force	N
*g*	Acceleration due to gravity	$\hbox {m\,s}^{-2}$
*K*	Empirical constant, see Eq. 12	–
*m*	Particle mass	kg
*p*	Polymer Cross exponent	–
*r*	Particle radius	m
Re	Reynolds number	–
${\text{Re}}_{\infty }$	Reynolds number calculated from $v_s$	–
*t*	Time	s
*T*	Temperature	°C
*U*	Free stream velocity	$\hbox {m\,s}^{-1}$
*v*	Particle velocity	$\hbox {m\,s}^{-1}$
$v_{ed}$	Viscoelastic-corrected velocity	$\hbox {m\,s}^{-1}$
$v_\infty$	Unbounded particle settling velocity	$\hbox {m\,s}^{-1}$
$v_m$	Measured particle velocity	$\hbox {m\,s}^{-1}$
$v_p$	Predicted particle velocity	$\hbox {m\,s}^{-1}$
$v_s$	Stokes’ velocity	$\hbox {m\,s}^{-1}$
$v_{sd}$	New Stokes’ velocity	$\hbox {m s}^{-1}$
$v_w$	Bounded particle settling velocity	$\hbox {m\,s}^{-1}$
$v_{wc}$	Wall-corrected settling velocity	$\hbox {m\,s}^{-1}$
$v_{wcd}$	New wall-corrected settling velocity	$\hbox {m\,s}^{-1}$
*W*	Distance to the wall	m
Wi	Weissenberg number	–
*x*	Distance	m
*z*	Distance travelled by particle between each frame	m
$\Delta$	Percentage difference	%
$\dot{\gamma }$	Shear-rate	$\hbox {s}^{-1}$
$\dot{\gamma }_d$	Shear-rate from diameter	$\hbox {s}^{-1}$
$\eta$	Apparent viscosity	Pa s
$\eta _a$	Average apparent viscosity	Pa s
$\eta _\infty$	Infinite shear apparent viscosity	Pa s
$\eta _0$	Zero shear apparent viscosity	Pa s
$\lambda$	Sphere-to-tube diameter ratio	–
$\lambda _c$	Critical sphere-to-tube diameter ratio	–
$\lambda _m$	Maxwell relaxation time	s
$\mu$	Newtonian viscosity	Pa s
$\rho _p$	Particle density	$\hbox {kg m}^{-3}$
$\rho _f$	Fluid density	$\hbox {kg m}^{-3}$
$\tau$	Shear-stress	Pa

A dash (–) indicates that the parameter is dimensionless.

Stokes’ Law is less applicable where the fluid in which the particle settles is non-Newtonian, especially where the viscosity is not constant and depends on the shear-rate applied. Non-Newtonian fluids are an important group of fluids in nature^4–8,27–32^ and in industry^9,10,33^, and there are a number of studies that consider the effects of non-Newtonian fluid rheology on particle settling^34–52^. However, there are substantially fewer studies on the effects of walls on particle settling in non-Newtonian fluids^18,22,25,26,53–55^ and even fewer studies of wall effects on settling when the fluid has both viscoelastic and shear-thinning properties. Zhang et al.^56^ considered the effects of walls on spheres settling in aqueous solutions of uncross-linked guar gum and polyacrylamide, both shear-thinning, viscoelastic, non-Newtonian fluids, and their experiments had a range of 0.02 $< \lambda<$ 0.9. They found that the shear-thinning properties of the fluids reduced the retardation effect on settling velocity, and were further weakened by the elastic effects of the viscoelastic polyacrylamide fluid. Subsequently, Song et al.^57^ used non-Newtonian, shear-thinning, viscoelastic fluids, to show that wall effects are reduced in shear-thinning fluids (relative to Newtonian fluids). Effects due to the viscoelastic properties of the fluid in their experiments were found to be minimal, and rather their study found a dependence on the wall correction factor with the sphere-to-tube diameter ratio and rheology model (Carreau) fit parameters. Jin and Chenevert^58^ calculated the settling velocity of a sphere in a shear-thinning viscoelastic fluid, based on an empirically established drag curve for the falling sphere. Malhotra and Sharma^53^ provide a means of accounting for both shear-thinning and viscoelastic effects in the presence of walls for power law fluids where $0.9\le n\le 1$. Other works consider the wall effect on viscoelastic fluids (Cho et al.^59^ and Chhabra et al.^60^), viscoplastic fluids (Atapattu et al.^61^), and power law fluids (Chhabra et al.^62^ and Lali et al.^18^) for example. Our work aims to expand the existing experimental data set and provide a more widely applicable correction for particles settling in a shear-thinning, viscoelastic fluid in the presence of wall effects.

Here, we present data from a set of experiments in Newtonian and non-Newtonian fluids using a range of different particle diameters. Our results indicate that (i) shear-thinning properties reduce the magnitude of wall effects by decreasing the distance over which the fluid is sheared. (ii) viscoelastic properties further reduce wall retardation effects and increase the drag on a particle in the high Weissenberg number regime (Wi $\gtrsim 1$) . (iii) the appropriate choice of the correct length-scale when calculating the shear-rate and related apparent viscosity, $\eta$, is imperative for accurate terminal velocity prediction, in addition to applying viscoelastic corrections using the Weissenberg number. These results have importance for improving settling velocity predictions in shear-thinning, viscoelastic, non-Newtonian fluids in the presence of walls, an area which lacks rigorous experimental data in the literature and has numerous real world applications.

## Background: Stokes’ law and wall effect corrections

This section gives an overview of the key equations and parameters required for calculating the terminal settling velocity of a solid particle within a fluid. The following subsections explain the derivation of Stokes’ Law, when it is applicable, and what corrections currently exist to account for boundary constrictions, such as the presence of walls that exert an influence on the terminal settling velocity.

### Terminal settling velocity

Stokes’ Law provides an empirical equation for calculating the settling velocity of a smooth, solid sphere falling in a Newtonian fluid (a fluid where the viscosity is independent of the shear-rate applied)^63^. Knowledge of the physical properties of both the particle and the host fluid are required to calculate the terminal settling velocity. Stokes’ Law assumes that the separation between the particle and any physical boundary (i.e., wall) is large enough that the particle settles under a laminar flow regime and is unaffected by shear against the wall. Stokes’ original equation^1^ defines the drag force, $F_D$, on a particle falling in a fluid as:

1 $$\begin{aligned} F_D = 3\pi \mu Ud \end{aligned}$$

where $\mu$ is the Newtonian viscosity of the fluid, *U* is the free stream velocity, and *d* is the diameter of the spherical particle. The derived equation for Stokes’ terminal velocity, $v_s$, is given by:

2 $$\begin{aligned} v_s = \frac{2r^2(\rho _p-\rho _f)g}{9\mu } \end{aligned}$$

where *r* is the radius of the particle, $\rho _p$ is the particle density, $\rho _f$ is the density of the fluid, and *g* is the acceleration due to gravity.

### The Reynolds number

A dimensionless group that describes the ratio of inertial to viscous forces, the Reynolds number is used to determine whether fluid flow is laminar or turbulent^4^. Equation 2 is only valid when Re < 1, where viscous forces dominate over inertial forces, and the regime is strictly laminar^64^. The Reynolds number is given by:

3 $$\begin{aligned} \textrm{Re} = \frac{\rho _fvd}{\mu } \end{aligned}$$

where *v* is the particle velocity. Note that the Stokes’ terminal settling velocity, $v_s$, and other velocities (denoted with a subscript in this paper) can be used in place of *v* to calculate their respective values. All experiments in this study have Reynolds numbers < 1 except for three experiments, which featured the largest settling velocities (see Table S6 in the Supplementary Information, Re numbers 1.51 to 1.80), for which Stokes’ Law may not be strictly valid. However, some studies suggest that Stokes’ Law is only valid when Re $<<$ 1^11,65,66^. Larger particles used in this study, which settle at higher velocities, enter the intermediate Reynolds number regime ( 1 $\lesssim$ Re $\lesssim$ 100)^67^. It may be observed that as Re becomes close to unity, particles also exhibit behaviour indicative of the intermediate Reynolds number regime, as the regime boundaries are only approximations and observable changes close to these boundary limits are expected.

### The drag coefficient

Particle settling rates have also been modelled as a function of the dimensionless variable drag coefficient, $C_d$, which is an expression of a particle’s resistance to flow in a fluid. $C_d$ values have been related to Re as^4^:

4 $$\begin{aligned} C_d = \frac{24}{\text {Re}}\left( 1+\frac{3}{16}\text {Re}\right) ; \quad \text {Re} <0.01 \end{aligned}$$

or

5 $$\begin{aligned} C_d = \frac{24}{\textrm{Re}}(1+0.1315\textrm{Re}^{(0.82-0.05\textrm{log}\mathrm {Re)}}); \quad 0.01< \text {Re} < 20 \end{aligned}$$

There is an extensive list of $C_d$ equations for higher Re values^4^, but this is outside the scope of this study given our experiments are focused on the low Re regimes.

### The wall effect

In situations where the particle can ’feel’ the wall of its container, an upward flow of fluid around the particle causes a reduction in settling velocity known as the wall effect^56^. This wall effect can be quantified by the wall factor, *f*:

6 $$\begin{aligned} f=\frac{v_w}{v_\infty } \end{aligned}$$

where $v_w$ is the terminal settling velocity of the sphere in a bounded fluid, and $v_\infty$ is the terminal velocity in an unbounded fluid. The wall factor can be expressed as a function of the Reynolds number and the sphere-to-tube diameter ratio, $\lambda$, where $\lambda = d/D$, and *D* is the diameter of the cylindrical tube. Previous works have proposed critical sphere-to-tube diameter ratios, $\lambda _c$, where wall retardation effects become significant. Lali et al.^18^ suggested a value of $\lambda _c$ $\simeq$ 0.05, whereas Slattery and Bird^68^ suggested $\lambda _c \simeq$ 0.1. For shear-thinning fluids, the value of $\lambda _c$ may be higher, as shear-thinning fluid rheologies are found to reduce the influence of the wall effect^53^.

Fidleris and Whitmore^2^ provide a ’wall-corrected’ equation for the terminal settling velocity of a spherical particle settling in a quiescent Newtonian fluid in a cylindrical tube for $\lambda < 0.9$:

7 $$\begin{aligned} f = \left( \frac{1-\lambda }{1-0.475\lambda }\right) ^4 \end{aligned}$$

covering the low Re regime. For the intermediate Reynolds number range, the wall correction factor for Newtonian fluids is given by Wham et al.^69^:

8 $$\begin{aligned} f=\frac{1+0.03708(0.5\text {Re}_\infty )^{A_0}}{[1+0.3708(0.5f\text {Re}_\infty )]^BC} \end{aligned}$$

where $\textrm{Re}_\infty$ is the Reynolds number calculated from Stokes’ velocity, $v_s$, and

9 $$\begin{aligned} A_0=1.514-0.1016\text {ln}(0.5\text {Re}_\infty ) \end{aligned}$$

10 $$\begin{aligned} B=1.514-0.1016\text {ln}(0.5f\text {Re}_\infty ) \end{aligned}$$

11 $$\begin{aligned} C=\frac{1-0.75857\lambda ^5}{1-K\lambda +2.0865\lambda ^3-1.7086\lambda ^5+0.72603\lambda ^6} \end{aligned}$$

and

12 $$\begin{aligned} K=0.6628+1.458\textrm{exp}(-0.028175f\textrm{Re}_\infty ) \end{aligned}$$

Equations 7 and 8 cover a satisfactory range of Reynolds numbers (Re $<100$) for this study and the high Reynolds number range (Re $>100$) will not be explored further here. For this the reader is referred to Chhabra and Patel^4^, who have recently summarised wall-corrective equations for all Reynolds number regimes.

Notwithstanding previous work, here, we seek to establish a more robust means of modelling wall effects on particle settling in non-Newtonian fluids that are both shear-thinning and viscoelastic.

## Methods

Our experimental campaign comprises fluid experiments on Newtonian and non-Newtonian fluids. The Newtonian fluid experiments were performed as validation checks, to ensure that our experimental method was capable of determining an accurate measurement of settling velocity in agreement with Stokes’ Law. The non-Newtonian fluids explore the consequences of fluid rheology (namely shear-thinning and viscoelasticity) on wall effects and the terminal settling velocity of particles.

### Fluid preparation

A sugar solution (hereby referred to as ’golden syrup’) manufactured by Tate and Lyle was chosen as the Newtonian fluid to perform particle drop experiments, as it has been widely demonstrated to exhibit a Newtonian rheology over the temperature and shear-rate range used in this study^70,71^. Two different solutions of golden syrup were prepared, a diluted solution of approximately 99 wt%, and a pure solution of 100 wt%. These are referred to as ’diluted golden syrup’ and ’pure golden syrup’ herein. Tap water was used as the diluting fluid, and the dilutions were left to settle in a glass cylindrical tube of diameter 50 mm and height 340 mm, into which the particles were later dropped, to allow for bubbles introduced during sample preparation to escape for a minimum of 24 hours. The shear-thinning non-Newtonian polymer used was Hydroxyethyl cellulose (hereafter referred to as ’HEC’), manufactured by the Dow Chemical Company with the trade name Cellosize$^{\textrm{TM}}$ and grade QP 52000H. Weight percentage, wt%, concentrations of 1.25, 1.5, and 1.75 were prepared for the following experiments by mixing HEC powder with tap water. For example, in a 1 wt% solution, 1 g of HEC powder was mixed with 99 g of water, or 2 g of powder with 198 g of water, and so forth. The powder was weighed using a mass balance accurate to ± 0.0005 g. The water mass was calculated from the water volume (1 ml of water = 1 g of water), which was approximated using a measuring jug. When the correct amount of both quantities were obtained, the powder was mixed slowly into the water to prevent the excess production of bubbles^72^. At the point where the powder was fully dissolved, the solution was poured into the glass cylindrical tube and left for a minimum of 24 hours so any bubbles that did form from mixing could be released from the liquid. This wait time varied between weight percentage solutions of HEC, as lower viscosity solutions required less time for all bubbles to escape.

### Fluid physical property determinations

To obtain the viscosity of the golden syrups and HEC concentrations, rotational rheometry was performed using an Anton Paar MCR 702e Rheometer. Approximately 0.1 ml of liquid was loaded onto a 30 mm radius, 60 mm diameter stainless steel Peltier base plate. Rotational rheometry was then performed using a stainless steel cone with a 15 mm radius, 30 mm diameter and a $\hbox {1}^{\circ }$ angle. For best measurement results, a perfect fill of liquid was achieved so there was no sample overspill or underfill within the measuring geometry. The Peltier base plate was set to the desired temperature, which was left for ten minutes to ensure thermal equilibrium between the plate and the fluid. Shear-rate sweeps of $\hbox {10}^{-3}$ $\hbox {s}^{-1}$ to $\hbox {10}^3$ $\hbox {s}^{-1}$ measured the shear-stress, with 10 measurement points per decade and a log distribution. This established a reliable shear-rate range from the Newtonian measurements to then apply to HEC fluid rheology property determinations.

For the golden syrup, fluid temperatures increased as subsequent particle drops were performed throughout the course of the day. It was thus necessary to perform multiple shear-rate sweeps at different temperatures to produce a relationship between temperature and viscosity. There was also a small temperature gradient between the top and bottom of the cylindrical tube, and this was taken into account when performing rheological measurements. Five repeats of one temperature were carried out to obtain a representative error, and then one measurement for each of the other temperatures was performed. The viscosity was calculated using $\mu =\tau /\dot{\gamma }$, where $\tau$ is the shear-stress and $\dot{\gamma }$ is the shear-rate. For the HEC experiments, there was negligible or no temperature gradient within the fluid due to the shorter fall times of the particles and lower fluid density, resulting in no requirement for temperature-viscosity interpolation. Thus rheometry measurements were performed directly at the same temperature as those recorded in the particle drop experiments. We used the same shear-rate sweep method applied to the golden syrups, with five repeats to quantify error. A Cross model was fitted to produce a shear-rate-apparent viscosity relationship, where $\eta =\tau /\dot{\gamma }$, is the apparent viscosity (see Eq. 14 and later subsection for the Cross model equation).

The fluid density at the experiment temperature was calculated using an Anton Paar DMA 4101 density meter. Three temperature ramps were carried out at 0.1 °C intervals for each fluid over the range of temperatures observed during the experiments, with an initial wait time of 10 minutes before the first measurement to ensure the fluid reached the starting temperature. For each temperature step, three determinations were taken, and the fluid was not measured until the desired temperature was reached. Before each ramp, the U-cell was filled with a new sample of fluid to avoid remeasuring any abnormalities in the aliquot of fluid. Both density and rheology measurements were carried out within a week of conducting the experiments, as the fluids experience degradation (e.g., dehydration) over time^72^.

### Particle drop experiments

Spherical steel particles were chosen for use in the experiments of diameters 3.14 mm, 4.76 mm, 7.90 mm, 10.30 mm, 11.96 mm, and 15.00 mm. The diameter of the particle was measured three times using digital vernier calipers accurate to ± 0.005 mm to find an average. The mass of each particle was measured three times using a mass balance accurate to ± 0.0005 g to find an average. The same six particles of each size were used throughout all the experiments for consistency. The density of the particle was calculated using *m*/*V*, with volume *V* calculated using $\frac{4}{3} \pi r^3$, and *r* being the radius of the particle. A full list of particle properties can be found in the Supplementary Information (Table S1). During the experiment, the temperature of the fluid was measured continuously by a digital thermometer accurate to ± 0.05 $^{\circ }\hbox {C}$ and a thermocouple wire submerged approximately 5 cm into the fluid. A stopwatch accurate to ± 0.005 s was placed next to the cylindrical tube to record the interval between each picture (or frame) taken by an Olympus OM-D E-M10 Mark IV digital camera. Each particle was placed into the liquid in the centre of the cylindrical tube and the fall subsequently recorded via a sequence of images. In experiments with small fall velocities, images were taken manually. When fall velocities were large, a video with a known frame rate of 30 frames per second was taken. A diagram of the experimental setup is shown in Figure S1 in the Supplementary Information. Interactions between the particle and the bottom of the tube slowed the particle from its terminal velocity, so these values were excluded from calculating the average settling velocity, along with velocities at the start of the fall where the particle had yet to reach its terminal velocity.

### Image analysis

The images captured in the experiments were imported into Fiji^73^, an image analysis software (and a variant of ImageJ^74^). To measure the distance moved by the particle, the very bottom of the particle was tracked between each successive frame (see Figure S2 in the Supplementary Information). This gave a number of pixels representative of the distance, which was converted to mm using a pixel-to-mm scale. This scale was calculated using the known particle diameter. The x- and y-displacement was then found between each successive frame. We used two different scales for calculating the x- and y-displacement to eliminate any visual distortion from the container geometry. This scale was accurate to ± 0.5 mm due to visual distortion from the cylindrical tube causing distances to change from the centre of the cylindrical tube to the edge. The total distance travelled by the particle between each frame was determined by using Pythagoras’ theorem with the x- and y-displacement. The velocity of the particle for each time interval was then calculated using *z*/*t*, with *z* the distance travelled between each frame, and time *t* between frames displayed by the stopwatch. Using the x-scale, the distance from the wall, *W*, on both the left and right side were measured, which could then be used along with the calculated velocity to obtain shear-rates at each point along the path taken by the particle using:

13 $$\begin{aligned} \dot{\gamma } = \frac{v_m}{W} \end{aligned}$$

where $v_m$ is the measured particle velocity and $\dot{\gamma }$ is the shear-rate. A left, right, and average shear-rate was calculated using the corresponding distances to the wall.

### Non-Newtonian rheological model fitting

We fit a Cross model to the HEC experimental data ($\dot{\gamma }$ vs $\eta$) based on the function:

14 $$\begin{aligned} \eta = \eta _{\infty }+\frac{\eta _0-\eta _{\infty }}{1+(c\dot{\gamma })^p} \end{aligned}$$

where $\eta$ is the apparent viscosity, $\eta _{\infty }$ is the infinite-shear apparent viscosity, $\eta _0$ is the zero-shear apparent viscosity, *c* is the polymer Cross constant, and *p* is the polymer Cross exponent. Equation and variables adopted by Jones et al.^72^ from Cross^75^. Because $\eta _{\infty }$ is very difficult to reach experimentally, $\eta _{\infty }$ was taken as the viscosity of water at the corresponding experiment temperature, as this is the lowest possible viscosity the fluid could have^72^. In producing the Cross models, all values with a shear-rate lower than 0.1 $\hbox {s}^{-1}$ were removed, as accurate values of viscosity were difficult to obtain experimentally at such low shear-rates. Newtonian fluid rheometry analysis showed that before this shear-rate, the viscosity value was not accurate and an artefact of start-up flow. Similarly, we restricted our runs to 100 $\hbox {s}^{-1}$ to avoid the effects of viscous heating in the sample. The Cross models provide a shear-rate-viscosity relationship. With the measured shear-rate from both the left and right distances to the container wall, the Cross model provides a specific viscosity for each point during the particles settling. Using Eq. 2, a Stokes’ settling velocity is calculated based on the measured parameters. Three different viscosities at each point on the settling path are calculated from the left, right, and average shear-rate, giving a minimum, maximum, and average Stokes’ velocity. We do this to compare the calculated Stokes’ velocities against our experimental results.

## Results

### Fluid physical properties

Prior to performing particle drop experiments in the non-Newtonian fluid, we verified our experimental design by a series of particle settling experiments in two Newtonian fluids (i.e., golden syrup). Rheological measurements were made to establish the Newtonian viscosity of pure golden syrup (PGS) and a 99 wt% diluted version of the same syrup (DGS) over a shear-rate range of $\hbox {10}^{-1}$ $\hbox {s}^{-1}$ to $\hbox {10}^2$ $\hbox {s}^{-1}$ (Figs. S3 and S4 in the Supplementary Information). The measurements were made at several temperatures to establish the temperature dependence of viscosity and an uncertainty based on five repeat measurements of a single temperature. For pure golden syrup (PGS), the temperature-dependent viscosity varied between 67.1 Pa s and 77.8 Pa s over the temperature range 19.2 $^{\circ }\hbox {C}$ to 19.6 $^{\circ }\hbox {C}$, and is modelled as:

15 $$\begin{aligned} \mu = -4.586T + 159.8 \end{aligned}$$

Density was measured over the same temperature range of 19.2 $^{\circ }\hbox {C}$ to 19.6 $^{\circ }\hbox {C}$, and varied between 1438.6 $\hbox {kg m}^{-3}$ and 1438.9 $\hbox {kg m}^{-3}$. The temperature-dependent viscosity of dilute golden syrup (DGS) is modelled as:

16 $$\begin{aligned} \mu = 0.1T^2 -4.99T + 79.978 \end{aligned}$$

and viscosity varied between 19.0 Pa s and 20.4 Pa s and density varied between 1424.98 $\hbox {kg m}^{-3}$ and 1425.3 $\hbox {kg m}^{-3}$, over the temperature range of 20.4 $^{\circ }\hbox {C}$ to 20.8 $^{\circ }\hbox {C}$.

**Figure 1:**
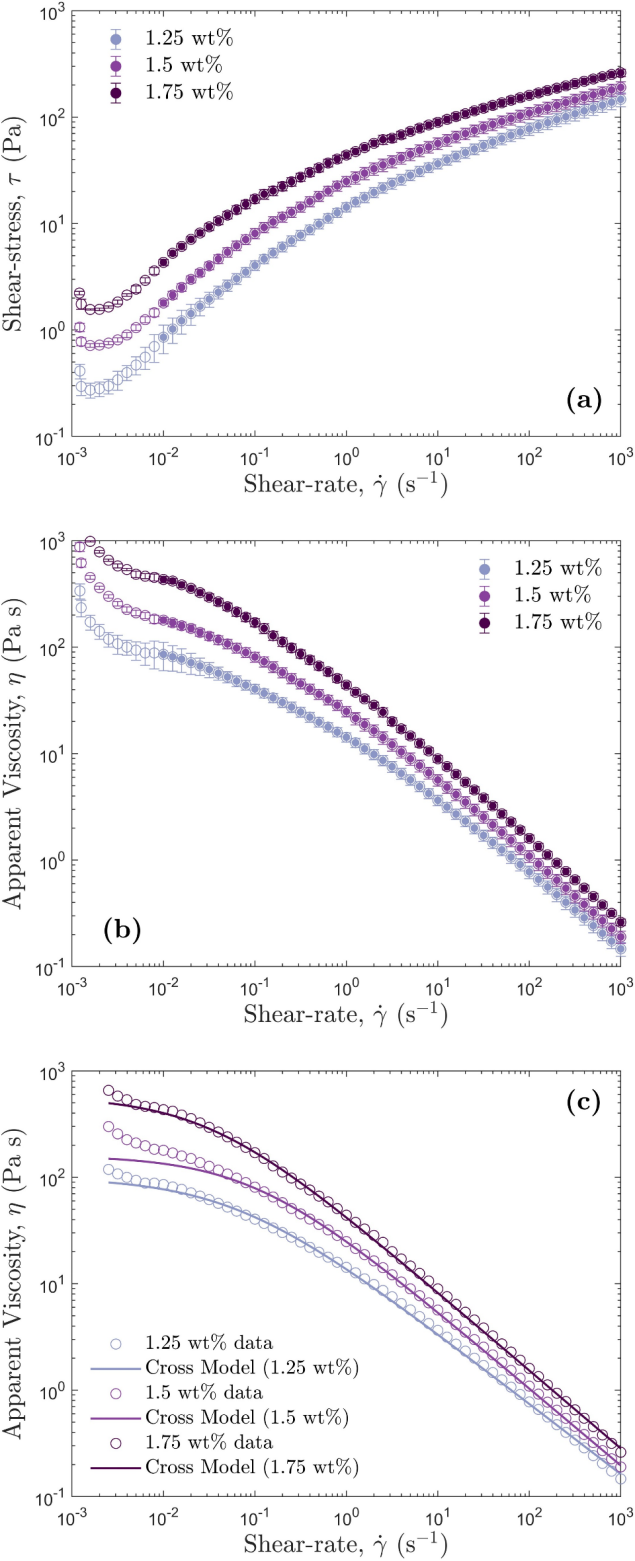
Rheological properties for three HEC fluids including: (**a**) Shear-rate shear-stress relationships. The filled data points indicate the data used in subsequent calculations. (**b**) Calculated shear-rate apparent viscosity relationships. (**c**) Cross model fits for each HEC fluid. Maximum and minimum Cross models fits based on error bars are reported in the Supplementary Information (Figures S5, S6, and S7).

**Figure 2:**
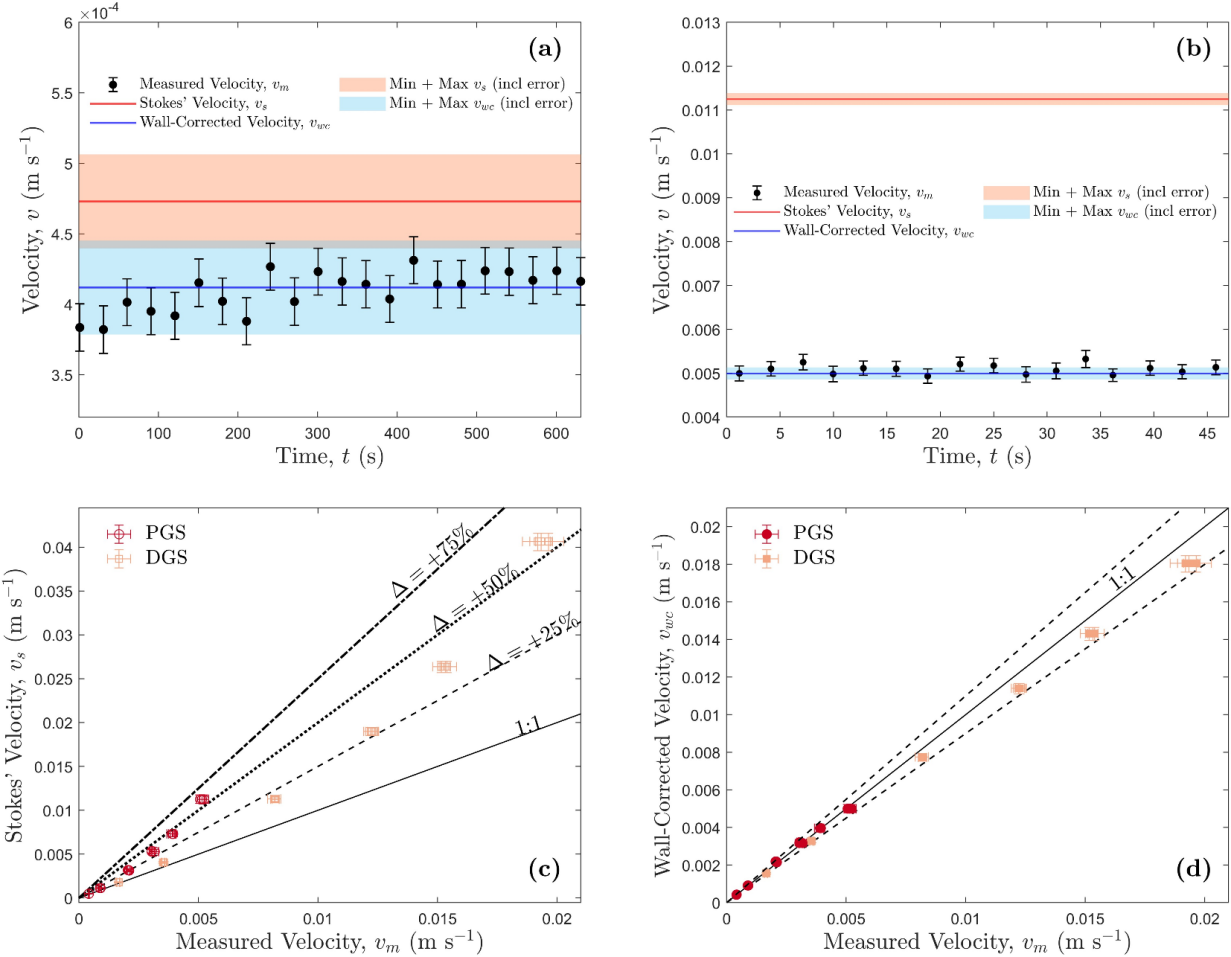
Experimental results for pure and diluted golden syrup particle drops. Error bars represent one standard deviation of uncertainty. (**a**) Experiment PGS-D1, an example of the smallest particle size. The measured particle settling velocity is plotted as a function of time, with the calculated Stokes’ and the wall-corrected settling velocity calculations shown by the red and blue solid lines, respectively. The shaded regions in the graph show the propagated error in the predicted velocities, $v_s$ and $v_{wc}$. Error from viscosity changes due to temperature fluctuations are smaller and thus not shown. (**b**) Experiment PGS-D16, an example of the largest particle size with the same symbology as in panel (**a**). (**c**) The predicted Stokes’ velocity compared to the measured velocity. The dashed lines represent different percentage deviations from the $v_s=v_m$, 1:1 line. (**d**) The predicted wall-corrected velocity compared to the measured velocity. The two dashed lines represent a $\pm \,10\%$ deviation from the $v_{wc}=v_m$, 1:1 line.

HEC has a shear-thinning rheology^72^, meaning that its viscosity is a function of shear-rate as well as temperature. We fit Cross models to viscosity shear-rate datasets (Fig.  1) over the shear-rate range of $\hbox {10}^{-1}$ $\hbox {s}^{-1}$ to $\hbox {10}^2$ $\hbox {s}^{-1}$, to calculate an average shear-rate viscosity relationship at a fixed temperature based on five repeat shear-rate sweeps. The resulting uncertainties in Cross model viscosity resulting from the repeat measurements were used to calculate an average, minimum, and maximum Stokes’ and wall-corrected settling velocity from the measured shear-rate. The maximum temperature fluctuations in the experiments were $\sim$ 0.1 $^{\circ }\hbox {C}$, causing insignificant variations in viscosity relative to the uncertainties on the Cross model values for viscosity. The density of HEC is consistent at a given temperature regardless of shear-rate, so the same method used for golden syrup was repeated for all HEC solutions. For 1.25 wt% HEC, the average $\eta _0= 100$ Pa s, and density was 1002.0 $\hbox {kg m}^{-3}$. For 1.5 wt% HEC, the average $\eta _0= 160$ Pa s, and density was 1003.5 $\hbox {kg m}^{-3}$. For 1.75 wt% HEC, the average $\eta _0= 575$ Pa s, and density was 1004.4 $\hbox {kg m}^{-3}$. Full tables of experimental fluid properties for golden syrup and HEC can be found in the Supplementary Information (Tables S2 and S3). A list of all Cross model parameters and fluid rheology properties for HEC can be found in Table S4 in the Supplementary Information.

### Particle drop results

**Figure 3:**
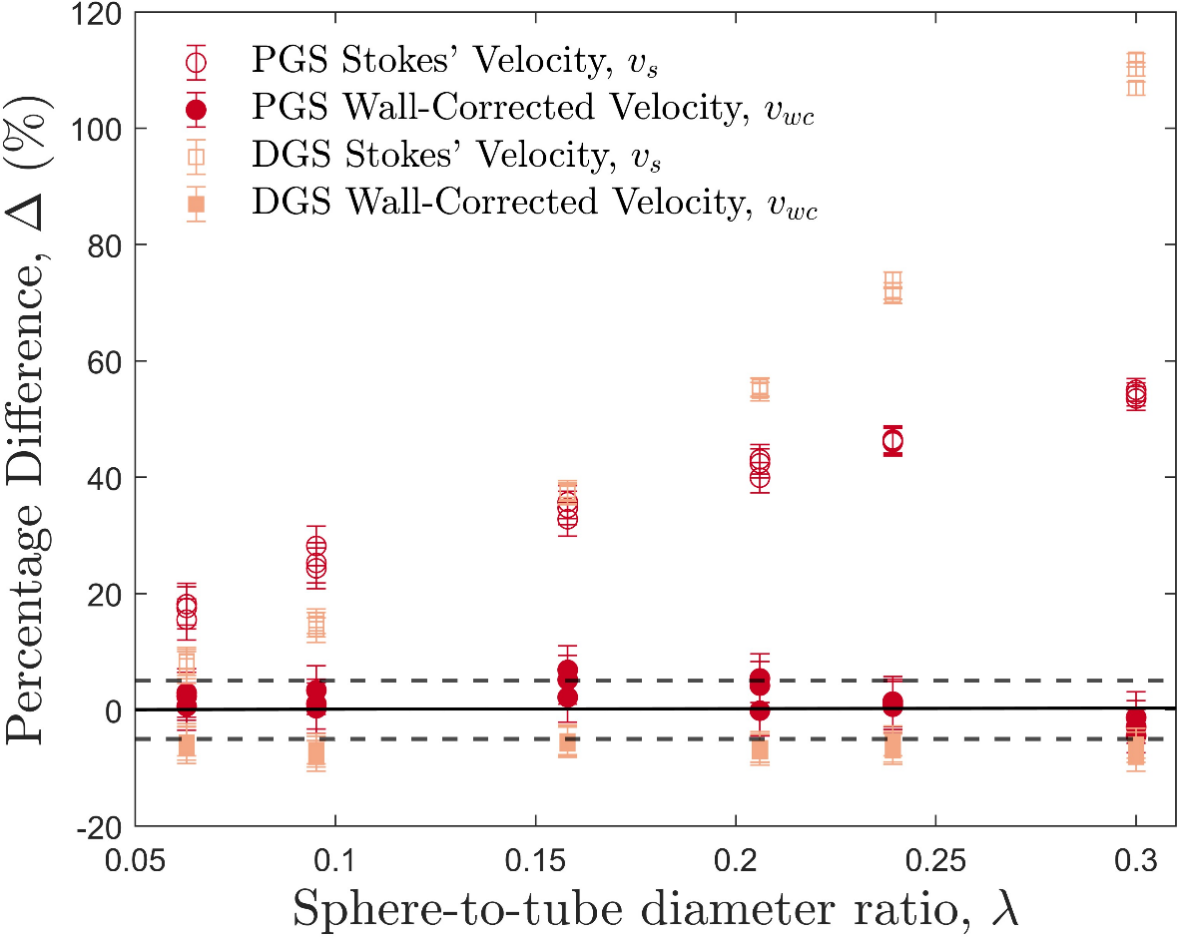
The percentage difference, $\Delta$, of the predicted velocities from the measured velocity, against the sphere-to-tube diameter ratio, $\lambda$, for all particle drop experiments using Newtonian fluids. The solid black line indicates a percentage difference of 0%, i.e., the predicted velocity matches the measured velocity, and the dashed black lines show a percentage difference of $\pm \,5\%$. Data points above the solid black line represent predicted velocities higher than the measured, and those below represent predicted velocities lower than the measured. Error bars represent one standard deviation of uncertainty.

The particle drop experiments in the Newtonian fluids produce terminal settling rates that are in excellent agreement with the predicted wall-corrected velocities. Two representative experiments are shown in Fig. 2a,b, with sphere-to-tube diameter ratios, $\lambda$, of 0.0628 and 0.3 respectively. The predicted Stokes’ velocity is higher than the measured velocity for the entire range of experiments, whereas the wall-corrected settling velocity shows very good agreement (Fig. 2c,d). The predicted Stokes’ velocity is much larger in 2b than 2a, due to the larger sphere-to-tube diameter ratio, $\lambda$, and subsequent increase in the magnitude of the wall effect. Figure 3 shows how the predicted velocities vary with $\lambda$. The percentage difference between the measured velocity and the predicted velocities, $\Delta$, was calculated using:

17 $$\begin{aligned} \Delta =\frac{v_p-v_m}{v_m}\times 100 \end{aligned}$$

where $v_p$ is the predicted velocity, either $v_s$ or $v_{wc}$. Between the region 0.0628 $< \lambda<$ 0.3, the disagreement between Stokes’ velocity and the predicted velocity increases with increasing $\lambda$, whereas the wall-corrected velocity deviates less than ± 10% from the measured velocity. The pure golden syrup measured velocities showed closer agreement with the wall-corrected velocities than the diluted syrup. This is likely to be attributed to the lower settling velocities of the pure golden syrup experiments, which reduce the measurement error associated with image processing. Percentage deviations between the measured velocity and the wall-corrected velocity did not exceed 6% for the pure golden syrup, with most experiments having less than a 5% deviation, and 13 of the 18 wall-corrected velocities are within error of the measured velocity. For the diluted syrup, deviations were consistently around 6% and 7%. From these results, it is clear that the wall-corrected settling velocity provides an accurate prediction for a particle settling in a Newtonian fluid. Therefore, it can be used as a comparison with the following HEC particle drops to see the effects of a non-Newtonian, shear-thinning, viscoelastic fluid on the settling velocity of a rigid, solid particle. A table of experimental results from all of the golden syrup particle drops can be found in the Supplementary Information (Table S5).

**Figure 4:**
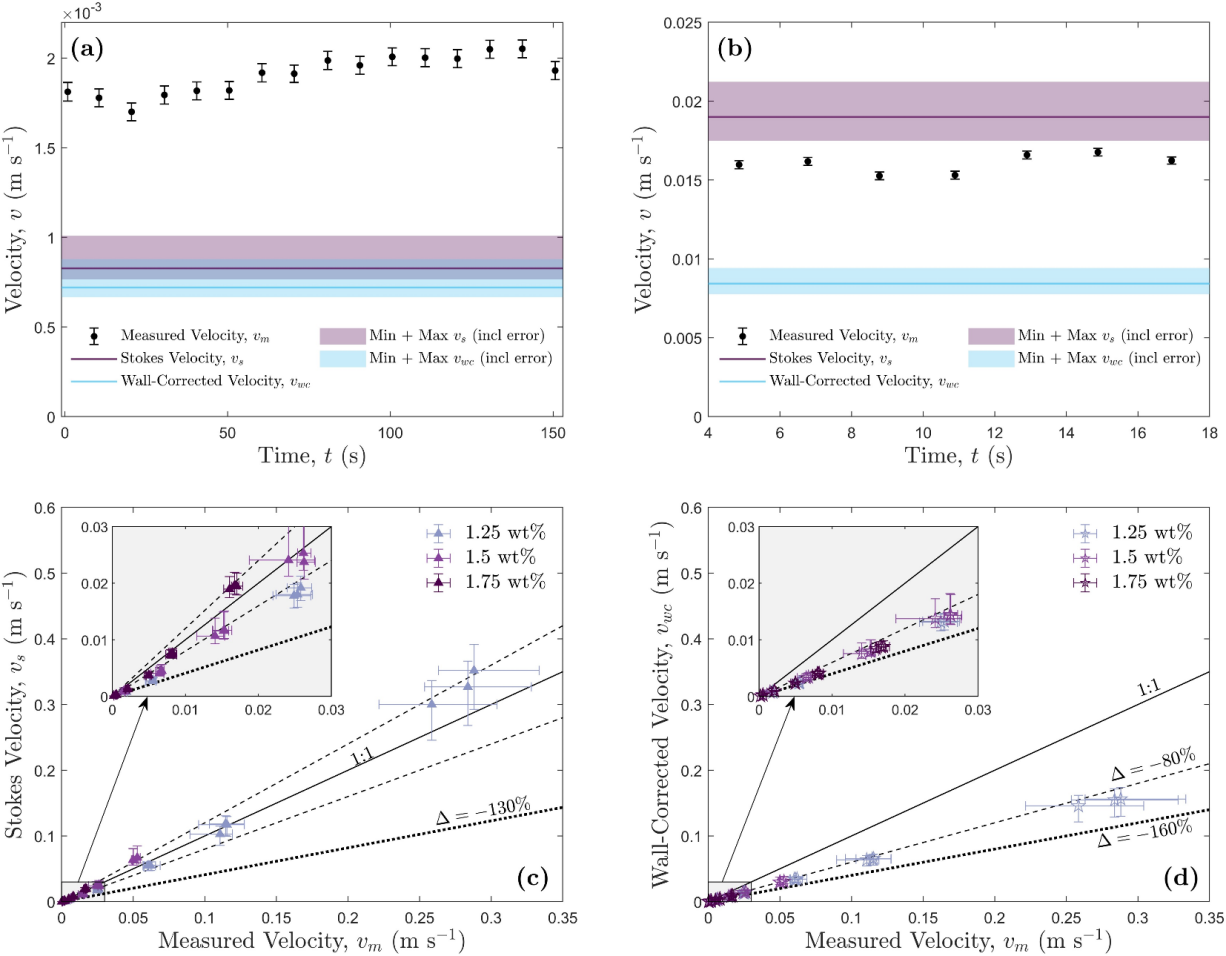
Experimental results for HEC particle drops. Error bars represent one standard deviation of uncertainty. (**a**) Results from experiment 1.25-D1. The measured particle settling velocity is plotted as a function of time, with the calculated Stokes’ and wall-corrected settling velocity represented by the purple and blue solid lines respectively. The shaded regions show the deviations in predicted Stokes’ and wall-corrected velocity due to shear-rate variations (i.e., the minimum and maximum Cross models). Error for the predicted velocities from measured viscosity repeats are smaller and thus not shown. (**b**) Results from experiment 1.75-D16 with the same symbology as in panel (**a**). (**c**) The predicted Stokes’ velocity compared to the measured velocity. The dashed lines represent different percentage deviations from the $v_{s}=v_m$, 1:1 line. The unlabelled dashed lines represent a percentage deviation of $\pm \,20\%$ from the 1:1 line. (**d**) The predicted wall-corrected velocity compared to the measured velocity. The dashed lines represent different percentage deviations from the $v_{wc}=v_m$, 1:1 line.

**Figure 5:**
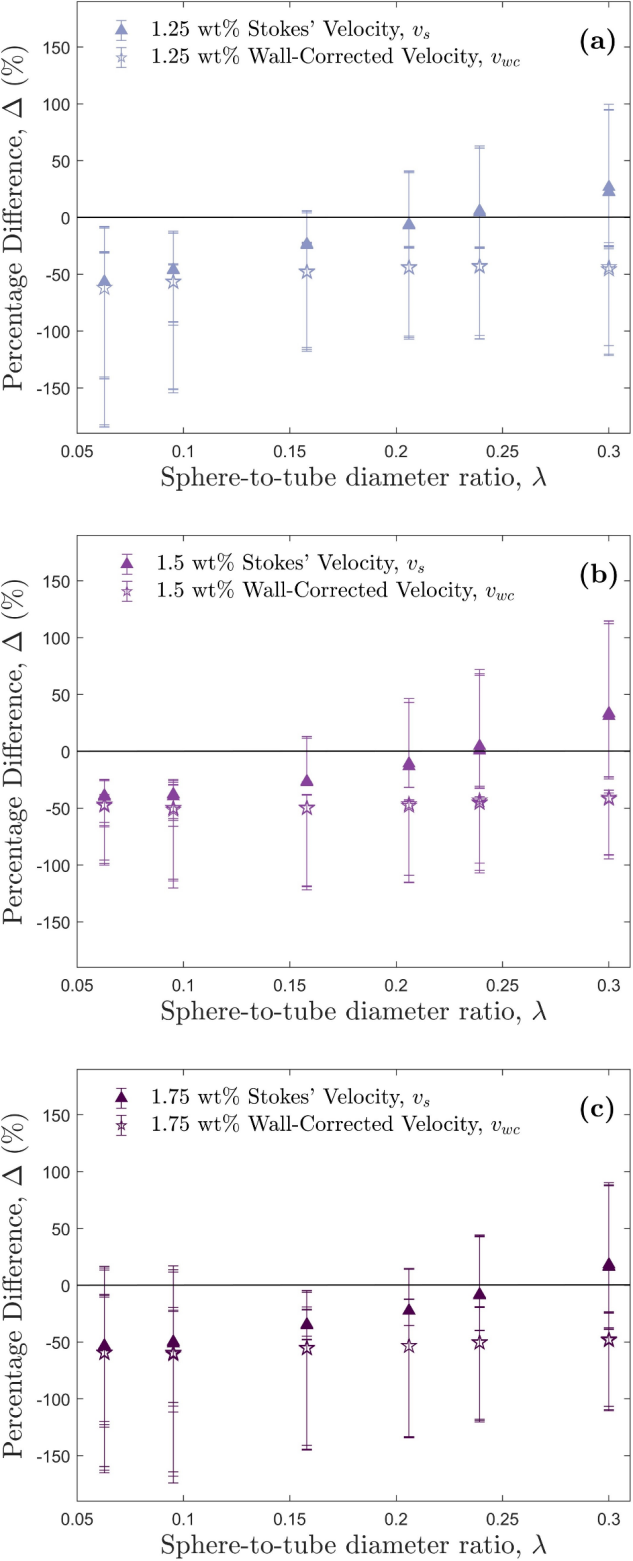
Percentage differences between predicted and measured velocities, $\Delta$, against the sphere-to-tube diameter ratio, $\lambda$, for (**a**) 1.25 wt%, (**b**) 1.5 wt%, and (**c**) 1.75 wt% HEC concentrations. The solid black line indicates a percentage difference of $0\%$, i.e., the predicted velocity matches the measured velocity. Data points above the solid black line represent predicted velocities higher than the measured, and those below represent predicted velocities lower than the measured. Error bars represent the minimum and maximum velocity differences calculated using the viscosities produced by the minimum and maximum Cross models.

For each HEC concentration (1.25 wt%, 1.5 wt%, and 1.75 wt%), 18 particle drops experiments were performed. Equation 13 was used to calculate the shear-rate, with viscosities then calculated using Eq. 14. This viscosity was then used in Eq. 2 to calculate Stokes’ terminal velocity. Lastly, this Stokes’ velocity was used in Eqs. 6 and 7 to calculate a subsequent wall-corrected velocity. Results from experiments 1.25-D1 and 1.25-D16, the smallest and largest particle drops respectively, are shown in Figure 4a,b. The measured velocity was much higher than the predicted velocities in Fig. 4a, whereas in Fig. 4b the measured velocity lies in between the two predicted/calculated values. Unlike the Newtonian golden syrup experiments, Stokes’ velocity provides a much closer approximation to the measured velocity compared to the wall-corrected velocity for all HEC concentrations (Fig. 4c,d) however, in most cases they still fail to match the observed velocities. Figure 5 shows that with an increase in $\lambda$, the predicted velocities get closer to the measured velocity up to $\lambda$ = 0.239, which has the minimum percentage difference ($\Delta$). The error bars shown in Fig. 5 represent the minimum and maximum velocities calculated using the minimum and maximum Cross models fitted to the rheological data. In the smaller shear-rate ranges, the difference in viscosity between the maximum and minimum model fits is much greater than in larger shear-rate ranges (see Figs. S5, S6, and S7 in the Supplementary Information). As a result, the larger error reported are not a result of experimental inconsistencies, but due to unavoidable viscosity variance in rheology measurements. The percentage difference between Stokes’ velocity, $v_s$, and the measured velocity, $v_m$, stays within error for all experiments where $\lambda$ > 0.158 (see Fig. 5). For the 1.75 wt% experiments, the wall-corrected velocity, $v_{wc}$, only comes within error of the measured velocities between $0.0628< \lambda < 0.0952$.

**Figure 6:**
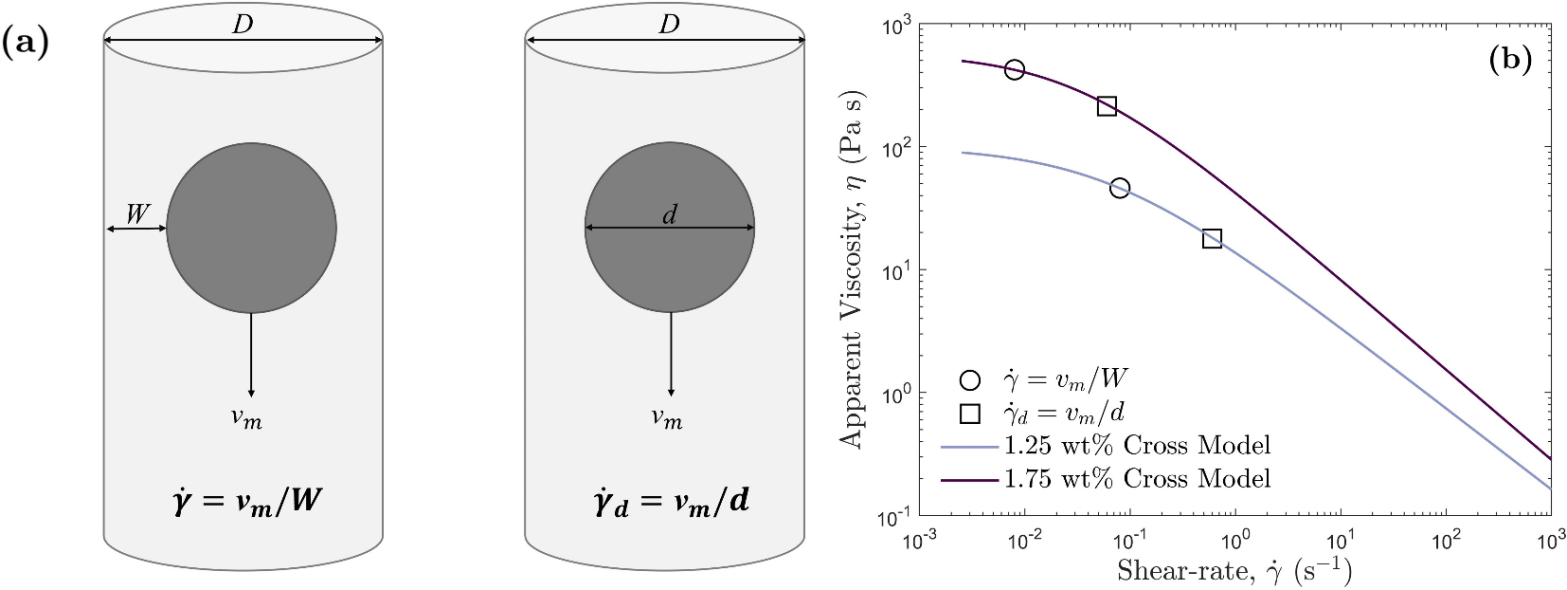
The calculation and comparison of shear-rates $\dot{\gamma }$ and $\dot{\gamma }_d$. (**a**) Diagrams displaying how the two different shear-rates are calculated based on two different length-scales. The cylindrical tube on the left shows the calculation of $\dot{\gamma }$, and the right shows the calculation of $\dot{\gamma }_d$. (**b**) Cross models of 1.25 wt% and 1.75 wt% HEC, with shear-rates from experiments 1.25-D1 and 1.75-D1 shown on the corresponding Cross model. The circles show values of $\dot{\gamma }$, and the squares show values of $\dot{\gamma }_d$. For $\dot{\gamma }_d$, the shear-distance is the diameter of the particle, *d*, rather than the distance to the wall, *W*. For small particles where $d < W$, $\dot{\gamma }_d > \dot{\gamma }$, which corresponds to a lower viscosity. When $d=W$, then $\dot{\gamma }_d=\dot{\gamma }$. When $d > W$, $\dot{\gamma }_d$ is no longer valid and the particle will be sheared by a distance *W* (i.e., experience shear-rate $\dot{\gamma }$).

Experiments using 1.25 wt% HEC had the highest measured settling velocities for each value of $\lambda$, as this concentration had the lowest apparent viscosity. This trend was consistent, with 1.75 wt% HEC having the lowest measured settling velocity and the highest apparent viscosity. HEC experiments with similar viscosities ($\sim$19.6 Pa s) to the diluted Newtonian experiments were 1.5-D13, 1.5-D14, and 1.5-D15 (the 1.5 wt% experiments with $\lambda$ values of 0.239), which had a viscosity of $\sim$20.6 Pa s. The measured settling velocity of experiments DGS-13, DGS-14, and DGS-15 (the diluted golden syrup experiments with $\lambda$ values of 0.239) were approximately 0.015 $\hbox {m s}^{-1}$, and the three 1.5 wt% HEC experiments had velocities of 0.026 $\hbox {m s}^{-1}$. Thus, for particle drops within 1.5 wt% HEC, measured velocities were approximately 1.7x larger than measured velocities for particle drops within the comparable diluted golden syrup, despite identical particle sizes and similar viscosities. The same is true when comparing the 1.75 wt% HEC to the pure golden syrup, sharing similar viscosities of 73 Pa s and 70.5 Pa s respectively (experiments 1.75-13 to 1.75-15, and PGS-D13 to PGS-D15 with the same $\lambda$ value). The measured velocities for 1.75 wt% HEC were approximately 2x larger than the pure golden syrup velocities. For identical particle and tube sizes, this difference in velocity must be attributed to the non-Newtonian properties of HEC, which will now be explored in the Discussion section. A table of experimental results from all of the HEC particle drops can be found in the Supplementary Information (Table S6).

## Discussion

In this section, the validity of Stokes’ terminal settling velocity equation is firstly assessed by exploring the Re$-C_d$ range of all experiments in this study. Once validity is established, differences between the Newtonian and non-Newtonian results are then discussed. Lastly, the effects of shear-thinning and viscoelasticity are analysed and corrections for the non-Newtonian fluids found.

### Stokes’ analysis for Newtonian fluids

Experiments in this study lie in the Re$-C_d$ range of $1.44 \times 10^{-6} <$ Re $< 1.80$, and $16.1< C_d < 1.67 \times 10^7$ (shown in Fig. S9 in the Supplementary Information). Apart from experiments 1.25-D16, 1.25-D17, and 1.25-D18 (experiments with the largest diameter particles and lowest apparent viscosity), all experiments fall within the low Reynolds number range. This means that, theoretically, Stokes’ terminal settling velocity should apply to all but three experiments in this study, and any deviations measured in the settling velocity can be attributed to other factors, such as the wall effect, shear-thinning, or viscoelastic fluid properties. The Newtonian golden syrup experiments show consistent agreement between the predicted wall-corrected velocity and the measured velocity within 5%. As $\lambda$ increases, the percentage difference between the predicted Stokes’ velocity and the measured velocity increases. This agrees with work by Song et al.^25^, which observes that with increasing $\lambda$ in Newtonian fluids, the drag on the particle due to the wall increases. The wall-corrected velocity accounts for the increasing drag, whereas the predicted Stokes’ does not. This is reflected in the large percentage deviations shown between the Stoke’s velocity and the measured velocity in Fig. 3.

The wall-corrected equation (Eqn. 7) provided by Fidleris and Whitmore^2^ shows that for particles settling in a bounded Newtonian fluid, the wall effect can be accounted for and Stokes’ equation adjusted to provide an accurate prediction for the terminal settling velocity when Re < 1. Wham et al.^69^ also found that wall effects diminished with increasing Re and decreasing $\lambda$. Furthermore, it has been suggested by Lali et al.^18^ that wall retardation effects are not present when $\lambda < 0.05$, but Slattery and Bird^68^ suggest a value of $\lambda < 0.1$. Here, for our Newtonian golden syrup experiments, the wall-corrected equation (i.e., Eqn. 7) provides a closer approximation than Stokes’ for the whole $\lambda$ range, indicating that wall retardation effects are present at all sphere-to-tube diameter ratios used in this study ($0.0628< \lambda < 0.3$). Furthermore, in our experiments Re $<< 1$, which concurs with the findings of Wham et al.^69^, that small Re values result in a larger influence of the wall effect.

### Corrections for non-Newtonian fluids

It has been previously observed that shear-thinning and viscoelastic effects reduce the wall effect on settling particles^56^. Our HEC experiments support this, as the Stokes’ settling velocity provides a better approximation to the measured velocity than the wall-corrected (Fig. 5), as Stokes’ assumes the particle is settling in an unbounded fluid, i.e., in absence of wall retardation effects. However, although a closer approximation, Stokes’ does not provide a consistent method of predicting particle terminal settling velocity in a non-Newtonian fluid, as we found that there were larger percentage deviations at lower sphere-to-tube diameter ratios, i.e., for the experiments where the distance to the wall was larger. Given the HEC fluid used in this study is both shear-thinning and viscoelastic, we need to consider the impact of both properties on the particle settling dynamics. Neither the original Stokes’ equation nor any of the wall-corrected equations provide a consistently accurate prediction for all of the measured velocities of the HEC experiments.

#### Accounting for shear-thinning effects

To account for the large deviations between the Stokes’ and measured settling velocity, it must be assumed that the particles are being sheared at a distance other than the distance between the particle edge and the wall of the container, *W*. Malhotra and Sharma^53^ find that shear-thinning effects reduce the drag on a particle leading to a higher settling velocity. Chhabra and Patel^4^ outline how experimental evidence suggests a smaller shear-rate than 2*v*/*d*, the general guide for Newtonian fluids, is experienced in shear-thinning fluids. This is an effect of the shear-thinning rheology^53,54,58^, and can be accounted for by finding a new length-scale that can be used to calculate the shear-rate that the fluid is experiencing, and thus will produce a settling velocity that is in agreement with observation. Consequently, we use a ’particle-induced’ shear-rate outlined by Bazilevskii^76^ for power law, shear-thinning fluids, where the shear-rate can be calculated as:

18 $$\begin{aligned} \dot{\gamma }_d= \frac{v_m}{d} \end{aligned}$$

**Figure 7:**
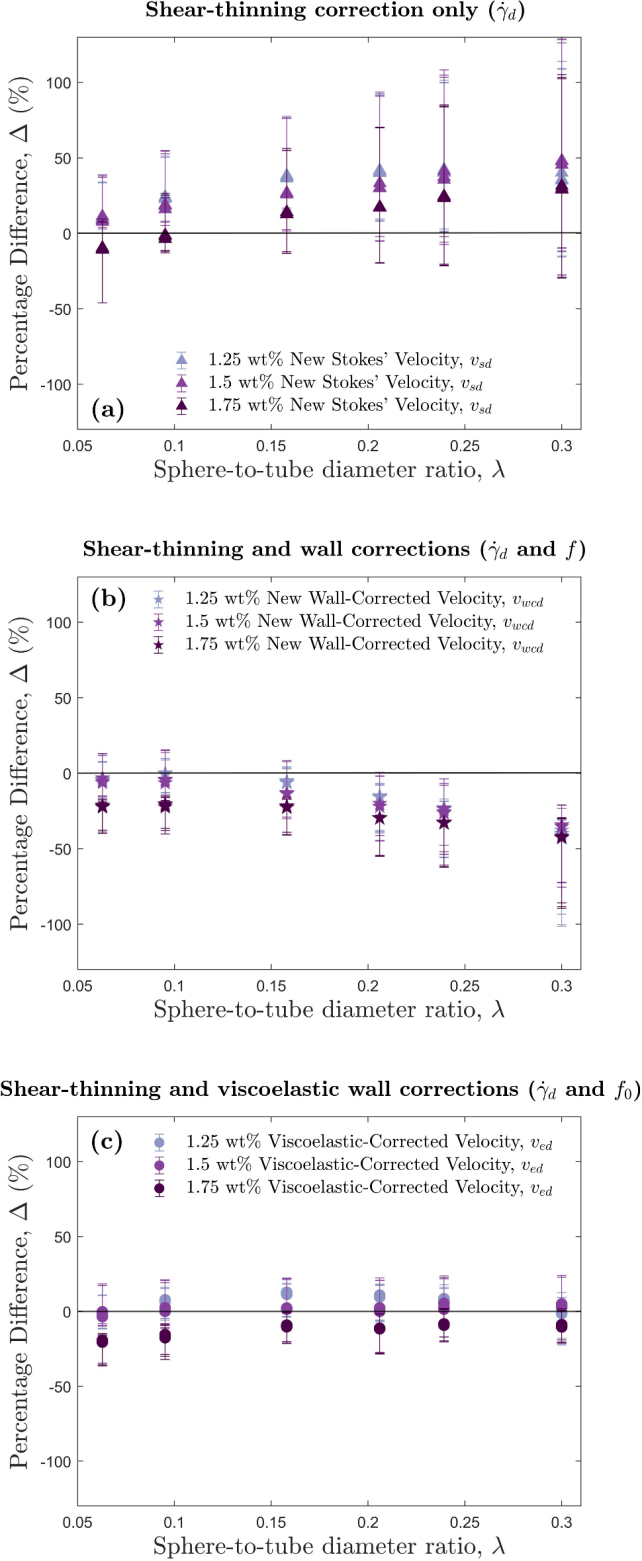
Percentage differences, $\Delta$, between predicted and measured velocities, against the sphere-to-tube diameter ratio, $\lambda$, for all HEC experiments. The solid black line indicates a percentage difference of $0\%$, i.e., the predicted velocity matches the measured velocity. Data points above the solid black line represent predicted velocities higher than the measured, and those below represent predicted velocities lower than the measured. All predicted velocities presented use $\dot{\gamma }_d$ to calculate the viscosity that is used in subsequent velocity calculations (see Fig. S10 in the Supplementary Information). Error bars represent the minimum and maximum velocity differences calculated from the viscosities produced by the minimum and maximum Cross models. (**a**) The shear-thinning correction only, represented by the new Stokes’ velocity, $v_{sd}$. (**b**) The shear-thinning and wall-corrected velocity, represented by $v_{wcd}$. (**c**) The shear-thinning and viscoelastic-corrected velocity, represented by $v_{ed}$.

Here, this shear-rate was calculated for each HEC experiment, shown in Fig. 6. When this shear-rate is used to calculate the apparent viscosity, Fig. 6b shows that due to the shear-thinning rheology of HEC, the viscosity can be significantly different even for small differences in shear-rate. The resulting percentage deviations from the measured velocity using the new shear-rate, $\dot{\gamma }_d$, and thus a shear-thinning correction is shown in Figs. 7a,b.

The use of $\dot{\gamma }_d$ provides a more consistent approximation to the measured velocity $v_m$, than the use of $\dot{\gamma }$ for at least $0.00628< \lambda < 0.3$ (i.e., the full range of $\lambda$ used in this study). Figure 7a shows the shear-thinning correction applied to Stokes’, and Fig. 7b shows the shear-thinning and wall-corrections applied to Stokes’. When $\lambda \gtrsim 0.1$ however, both $v_{sd}$ and $v_{wcd}$ (Fig. 7a,b) show increasing deviation from the measured velocity $v_m$. The deviations in $v_{wcd}$ from $v_m$ (Fig. 7b) imply that the shear-thinning properties of HEC reduce wall retardation effects, which agrees with observations by Zhang et al.^56^.The shear-thinning properties of HEC change the length-scale of the region of fluid sheared by the settling particle. When the diameter of the particle exceeds the distance between the particle and the wall, *W*, the shear length-scale is restricted to *W*. However, this was not the case for any of our particles, so we can assume all were sheared by a distance of *d*, and thus, the shear-thinning correction using $\dot{\gamma }_d$ is appropriate for all our experiments. However, deviations from the measured velocity $v_m$, are still observed when applying the shear-thinning correction without the wall correction (Fig. 7a), indicating that shear-thinning effects are not the only influence on settling velocity. Therefore, we must also examine the viscoelastic effects of our HEC polymer.

#### Accounting for viscoelastic effects

The viscoelastic properties of a fluid have been found to reduce the influence of the wall effect and either increase or decrease the drag on a falling particle^4,53,56,77,78^. Having accounted for the shear-thinning effects through $\dot{\gamma }_d$ vs $\dot{\gamma }$, it is now important to discern the impact that viscoelasticity has on the settling velocity in the HEC particle drop experiments. A dimensionless number that quantifies the viscoelastic effects of a fluid is the Weissenberg number^79^. The Weissenberg number is defined as:

19 $$\begin{aligned} \textrm{Wi}=2\lambda _m\dot{\gamma }\end{aligned}$$

where $\lambda _m$ is the relaxation time of the fluid. The relaxation times for our HEC concentrations are 1.3 s, 2.5 s, and 4 s, for the 1.25 wt%, 1.5 wt%, and 1.75 wt% concentrations, respectively. These values were taken from Jones et al.^72^, which used the exact same grade HEC and concentrations as used in this study. The Weissenberg numbers in this study, calculated using $\dot{\gamma }_d$, range from 0.5 $\lesssim$ Wi $\lesssim$ 50. A full list of Wi numbers for each experiment can be found in Table S7 in the Supplementary information.

The influence of viscoelasticity on the drag experienced by a particle depends on the Weissenberg number. In the low Wi regime, where Wi $\lesssim 1$, drag reductions leading to faster settling have been observed^26,56^, whereas in the higher Wi regime where Wi $\gtrsim 1$, an increase in drag leading to slower settling has been observed^53,80–83^. Chhabra and Patel^4^ provide an equation for estimating the viscoelastic wall correction factor, $f_0$, in viscoelastic fluids:

20 $$\begin{aligned} f_0=1-0.94\lambda ^{0.8}(\text {Wi})^{-0.073} \end{aligned}$$

valid in the range $\lambda \le 0.5$, $0.02 \le$ Wi $\le 11$, and Re < 1. We firstly applied the shear-thinning correction (i.e., the appropriate use of $\dot{\gamma }_d$ vs $\dot{\gamma }$) and then applied this viscoelastic correction (Eq. 20) to our experiments. The results of this are shown in Fig. 7c. For all values of $\lambda$ in this study, the combination of both shear-thinning and viscoelastic corrections provides the best approximation to measured velocities. For the 1.25 and 1.5 wt$\%$ experiments, all predicted velocities were within error of the measured velocity. For 1.75 wt$\%$ experiments, the smaller $\lambda$ value experiments were very close to being within error, but the viscoelastic effects may have been negligible in these settling scenarios due to the very small Reynolds numbers. This is demonstrated in the 1.75 wt$\%$ experiments by the similar $\Delta$ values for small $\lambda$ in Fig. 7b,c. In summary, we consider the application of both shear-thinning and viscoelastic corrections to be the best method of approximating the settling velocity of all settling scenarios in this study. Even though 12 of our 54 HEC experiments had Wi $> 11$, we find that Eq. 20 still provides a good correction for our data. By first applying the shear-thinning correction accounting for the smaller length scale over which the fluid is sheared, *d*, and then the viscoelastic wall correction factor, $f_0$, we have accurately predicted the settling velocity of spheres in a shear-thinning, viscoelastic fluid.

## Conclusions

For sphere-to-tube diameter ratios $0.0628< \lambda < 0.3$ (i.e., the full range of $\lambda$ used in this study), a shear-rate calculated using the particle diameter, *d*, as the length-scale, $\dot{\gamma }_d = v_m/d$, is identified as a closer approximation to shear-rates experienced by the fluid when solid, smooth, spherical particles settle in a shear-thinning, viscoelastic, non-Newtonian fluid. Using the corresponding apparent viscosity, Stokes’ terminal settling velocity is calculated. A viscoelastic wall correction factor is then applied, which shows good agreement with measured settling velocities. By comparing the calculated velocities before and after our shear-thinning and viscoelastic corrections, it is clear that both these properties influence particle settling dynamics. The shear-thinning properties increase the settling velocity by reducing the localised region of fluid that is sheared by the settling particle, resulting in a lower apparent viscosity. The viscoelastic properties in the higher Weissenberg number regime increase the drag on the particle, resulting in a decrease in settling velocity. In comparison with the conventional wall-corrected Stokes’ velocity (Fig. 5), the shear-thinning and viscoelastic-corrected velocities are around 50$\%$ larger. From this, we can conclude that the shear-thinning properties of HEC have a larger influence on the settling dynamics compared to the viscoelastic properties, due to the corrected velocities being larger than those predicted by Stokes’.

## Supplementary Information

Supplementary Information 1.

Supplementary Information 2.

Supplementary Information 3.

## Data Availability

All data generated or analysed during this study are included in this published article (and its Supplementary Information files).
