# Supplementary material for: Particle settling in a shear-thinning, viscoelastic fluid in the presence of wall effects
Source: Sci Rep. 2025 Feb 6;15:4482. doi: 10.1038/s41598-025-87742-w (PMC11802780; doi:10.1038/s41598-025-87742-w)
Supplement: Supplementary file 1 — Supplementary Information 1. [file 41598_2025_87742_MOESM1_ESM.pdf]

Supplementary Information to Accompany: Particle settling in a shear-thinning, viscoelastic fluid in the presence of wall-effects

Jodie Whorton, Thomas J. Jones, James K. Russell

Description of Additional Supplementary Files

File name: Supplementary Data 1.xlsx
Description: An excel file containing all of the raw rheological data, with a select few measurements shown in Figure S3 and Figure 1. The file contains five excel sheets corresponding to the five different fluid dilutions used in this study. "PGS" and "DGS" corresponding to pure golden syrup and diluted golden syrup respectively, and "1_25_HEC", "1_5_HEC", and "1_75_HEC" corresponding to the 1.25 wt%, 1.5 wt%, and 1.75 wt% dilutions of HEC respectively.

File name: Supplementary Data 2.xlsx
Description: An excel file containing all of the raw experimental data shown in Figures 2, 3, 4, and 5, and Tables S5 and S6. The file contains 90 excel sheets, each labelled with the corresponding experiment number reported in Tables S5 and S6.

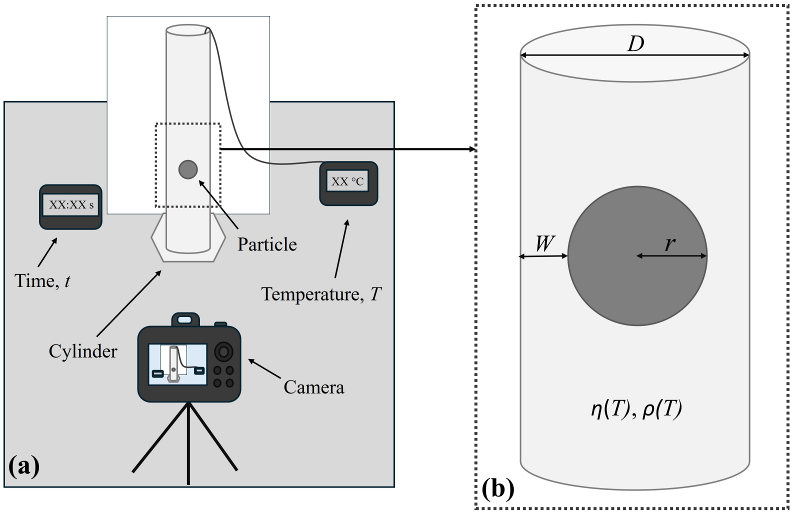

Figure S1. The experimental set up used in this study and the measured parameters. (**a**) An expanded view of the experimental setup. (**b**) A detailed view of the particle with radius *r* falling in the cylinder with a diameter *D* and distance to the wall *W*. The fluid has a temperature-dependent viscosity $\eta(T)$ and density $\rho(T)$.

Table S1. Particle physical properties of all spherical steel particles used in the particle drop experiments. Reported are the diameters and masses, both measured and averaged from three repeats. The particle density was calculated using the measured mass, and calculated spherical volume using the measured diameter (radius).

Particle diameter, d (m)	Particle mass, m (kg)	Particle density, ρ_p (kg m^{-3})
0.00314	0.00013	8019.7
0.00476	0.000444	7862.6
0.0079	0.002042	7910.0
0.0103	0.004487	7842.3
0.01196	0.007171	8005.5
0.015	0.01391	7871.4

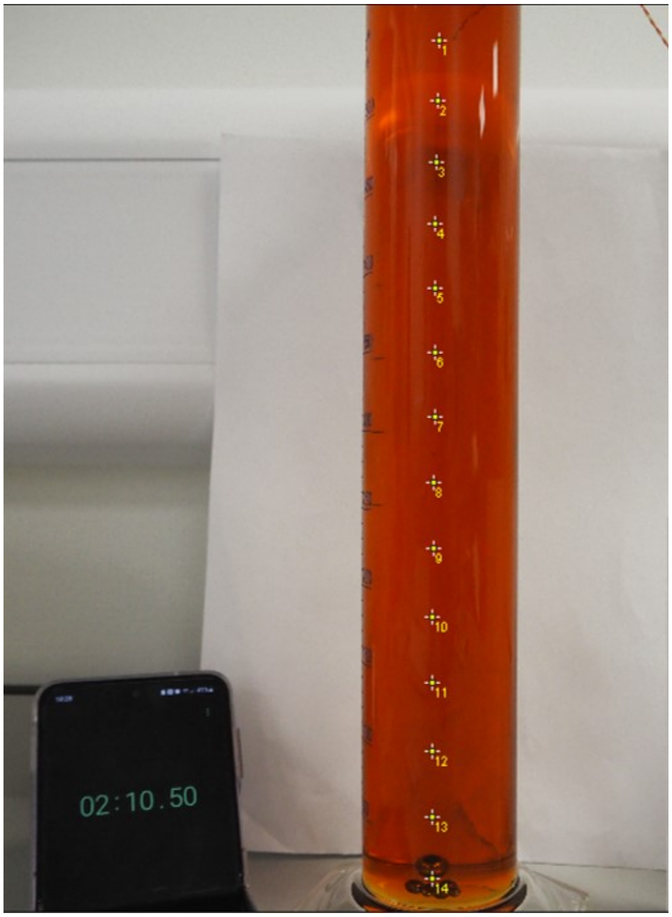

Figure S2. The image analysis process of a representative particle drop experiment. Each number point indicates the position of the bottom of the particle at an individual frame. The stopwatch to the left shows the total time of the captured experiment. The particle can be seen at the number 14 point, just before it touches the bottom of the cylinder.

Table S2. Fluid physical properties for pure (PGS) and diluted (DGS) golden syrup experiments. Reported are the experiment number, fluid temperature, Newtonian viscosity, and the fluid density.

Experiment number	Fluid temperature, T (°C)	Viscosity, μ (Pa s)	Fluid density, ρ_f (kg m^{-3})
PGS-D1	19.2	74.8	1438.9
PGS-D2	19.2	71.8	1438.9
PGS-D3	19.3	71.6	1438.7
PGS-D4	19.4	70.4	1438.7
PGS-D5	19.4	70.4	1438.7
PGS-D6	19.4	70.4	1438.7
PGS-D7	19.4	70.4	1438.7
PGS-D8	19.5	69.2	1438.6
PGS-D9	19.5	69.2	1438.6
PGS-D10	19.5	69.2	1438.6
PGS-D11	19.6	70.1	1438.6
PGS-D12	19.6	70.1	1438.6
PGS-D13	19.6	70.1	1438.6
PGS-D14	19.6	70.1	1438.6
PGS-D15	19.6	70.1	1438.6
PGS-D16	19.6	70.1	1438.6
PGS-D17	19.6	70.1	1438.6
PGS-D18	19.6	70.1	1438.6
DGS-D1	20.4	19.8	1425.3
DGS-D2	20.4	19.8	1425.3
DGS-D3	20.5	19.7	1425.2
DGS-D4	20.6	19.6	1425.2
DGS-D5	20.6	19.6	1425.2
DGS-D6	20.6	19.6	1425.2
DGS-D7	20.6	19.6	1425.2
DGS-D8	20.6	19.6	1425.2
DGS-D9	20.7	19.5	1425.1
DGS-D10	20.7	19.5	1425.1
DGS-D11	20.7	19.5	1425.1
DGS-D12	20.7	19.5	1425.1
DGS-D13	20.8	19.5	1425.0
DGS-D14	20.8	19.5	1425.0
DGS-D15	20.8	19.5	1425.0
DGS-D16	20.8	19.5	1425.0
DGS-D17	20.8	19.5	1425.0
DGS-D18	20.8	19.5	1425.0

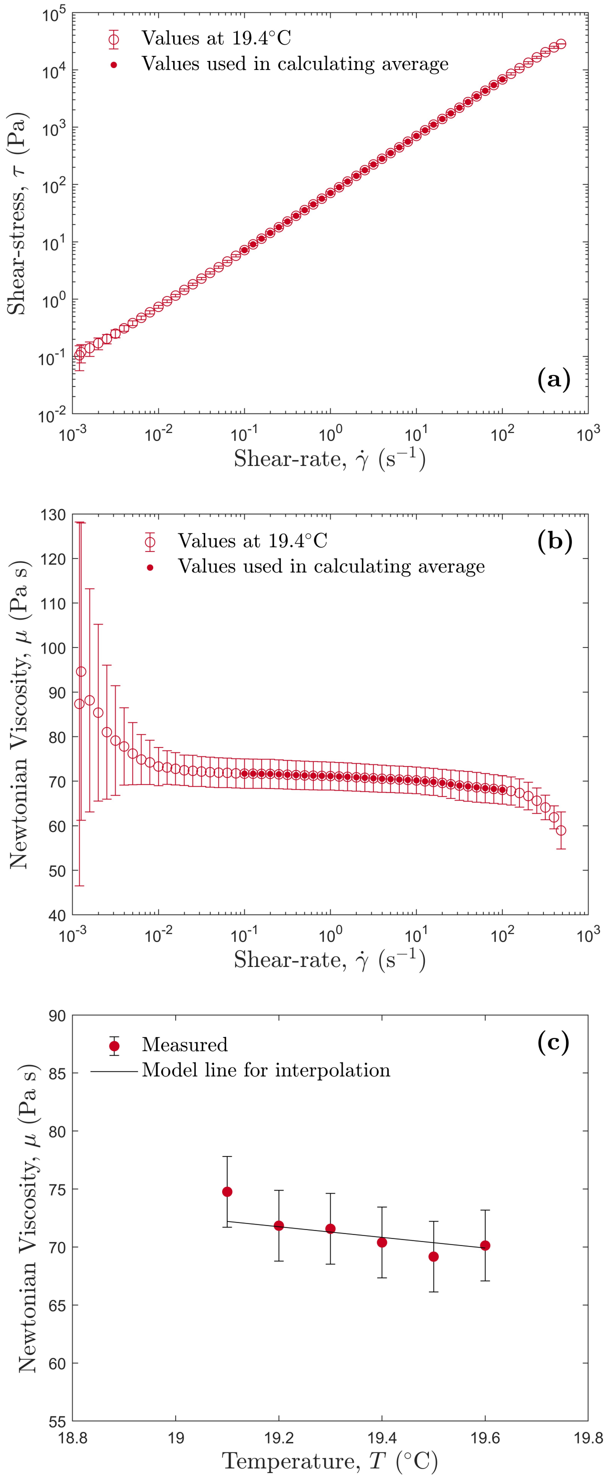

Figure S3. Rheological measurements for pure golden syrup. Error bars represent one standard deviation of uncertainty. **(a)** The average of five repeat shear-rate sweep measurements at 19.4°C, showing the relationship between shear-rate and shear-stress. **(b)** Using data from (a), the relationship between shear-rate and viscosity. **(c)** The relationship between temperature and viscosity.

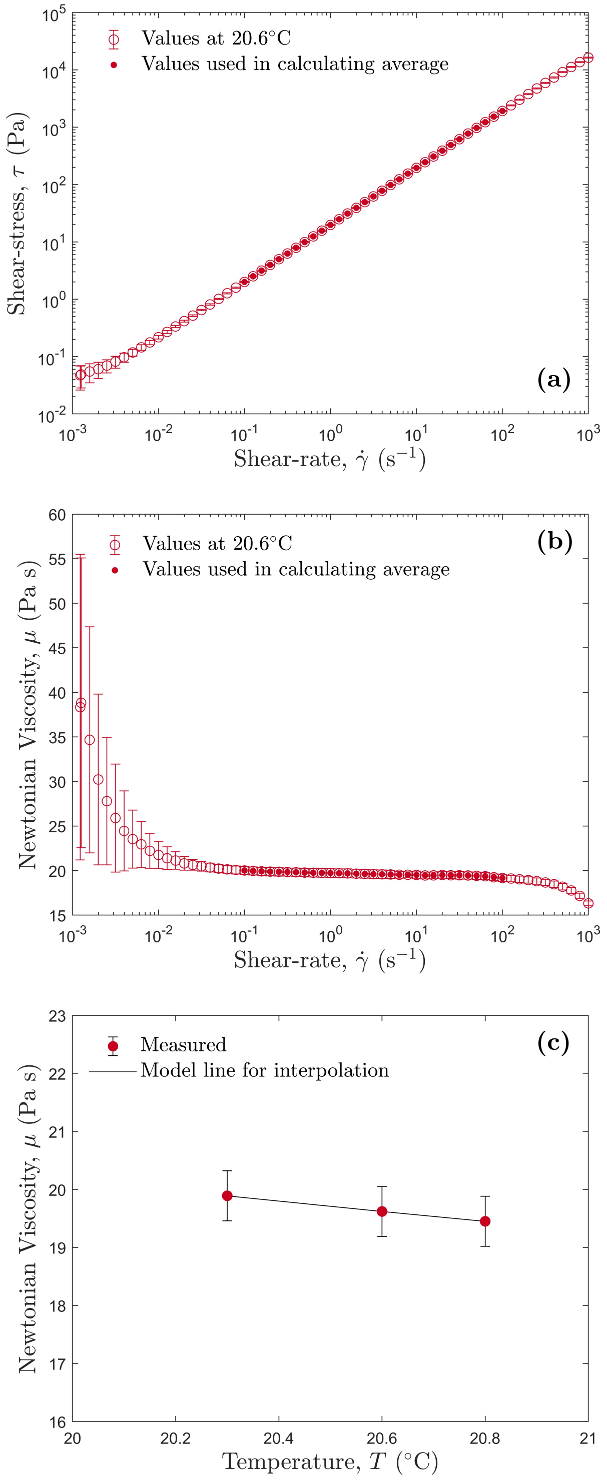

Figure S4. Rheological properties of diluted golden syrup. Error bars represent one standard deviation of uncertainty. (**a**) The average of five repeat shear-rate sweep measurements at 20.6°C, showing the linear relationship between shear-rate and shear-stress. (**b**) Using data from (a), the relationship between shear-rate and viscosity. (**c**) The relationship between temperature and viscosity, with the measured values providing a fit for the interpolated values. Equivalent data for pure golden syrup is presented in Figure S4.

Table S3. Fluid physical properties for all HEC experiments. Reported are the experiment numbers, the fluid temperature, apparent viscosity average from the shear-rate calculated using the distance to the wall, apparent viscosity average from the shear-rate calculated using the particle diameter, and the fluid density.

Experiment number	Experiment temperature, T (°C)	Viscosity average (Pa s) ($\dot{\gamma} = v_m/W$)	Viscosity average, (Pa s) ($\dot{\gamma} = v_m/d$)	Fluid density, ρ_f (kg m^{-3})
1.25-D1	21.1	45.6	18.1	1002.0
1.25-D2	21.1	45.9	18.3	1002.0
1.25-D3	21.1	45.6	18.1	1002.0
1.25-D4	21.1	28.4	12.4	1002.0
1.25-D5	21.1	28.8	12.6	1002.0
1.25-D6	21.1	28.7	12.5	1002.0
1.25-D7	21.1	12.0	6.68	1002.0
1.25-D8	21.1	12.2	6.76	1002.0
1.25-D9	21.1	12.3	6.84	1002.0
1.25-D10	21.1	7.0	4.7	1002.0
1.25-D11	21.1	6.9	4.61	1002.0
1.25-D12	21.1	6.9	4.56	1002.0
1.25-D13	21.1	4.7	3.48	1002.0
1.25-D14	21.1	4.5	3.39	1002.0
1.25-D15	21.1	4.6	3.40	1002.0
1.25-D16	21.1	2.4	2.20	1002.0
1.25-D17	21.1	2.6	2.33	1002.0
1.25-D18	21.1	2.4	2.17	1002.0
1.5-D1	20.3	118.5	63.9	1003.5
1.5-D2	20.3	119.7	65.5	1003.5
1.5-D3	20.3	119.4	65.0	1003.5
1.5-D4	20.3	92.1	48.9	1003.5
1.5-D5	20.3	92.0	48.8	1003.5
1.5-D6	20.3	89.7	46.8	1003.5
1.5-D7	20.3	48.7	28.3	1003.5
1.5-D8	20.3	47.8	27.7	1003.5
1.5-D9	20.3	49.6	29.0	1003.5
1.5-D10	20.3	31.7	21.3	1003.5
1.5-D11	20.3	29.8	19.9	1003.5
1.5-D12	20.3	30.0	20.0	1003.5
1.5-D13	20.3	20.5	15.2	1003.5
1.5-D14	20.3	20.7	15.4	1003.5
1.5-D15	20.3	21.7	16.1	1003.5
1.5-D16	20.3	12.6	11.4	1003.5
1.5-D17	20.3	12.7	11.4	1003.5
1.5-D18	20.3	12.2	11.0	1003.5
1.75-D1	19.0	417.4	217	1004.4
1.75-D2	19.0	416.5	216	1004.4
1.75-D3	19.0	418.7	218	1004.4
1.75-D4	19.0	318.9	164	1004.4
1.75-D5	19.0	316.9	162	1004.4
1.75-D6	19.0	313.6	159	1004.4
1.75-D7	19.0	171.2	98.4	1004.4
1.75-D8	19.0	174.3	100.6	1004.4
1.75-D9	19.0	170.9	98.2	1004.4
1.75-D10	19.0	102.5	68.0	1004.4
1.75-D11	19.0	102.9	68.2	1004.4
1.75-D12	19.0	102.4	67.9	1004.4
1.75-D13	19.0	73.7	54.4	1004.4
1.75-D14	19.0	72.9	53.8	1004.4
1.75-D15	19.0	74.4	54.9	1004.4
1.75-D16	19.0	44.4	39.9	1004.4
1.75-D17	19.0	42.6	38.3	1004.4
1.75-D18	19.0	43.1	38.8	1004.4

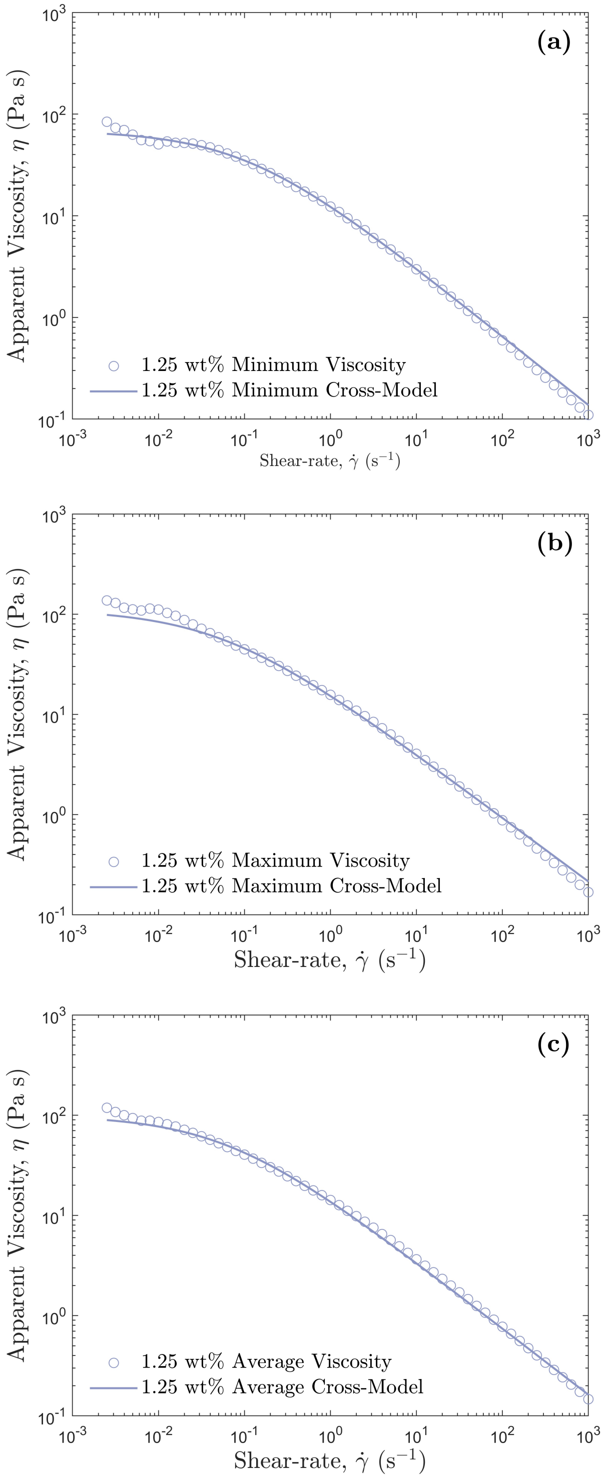

Figure S5. The range in Cross models produced for 1.25 wt% HEC. **(a)** Minimum Cross model. **(b)** Maximum Cross model. **(c)** Average Cross model.

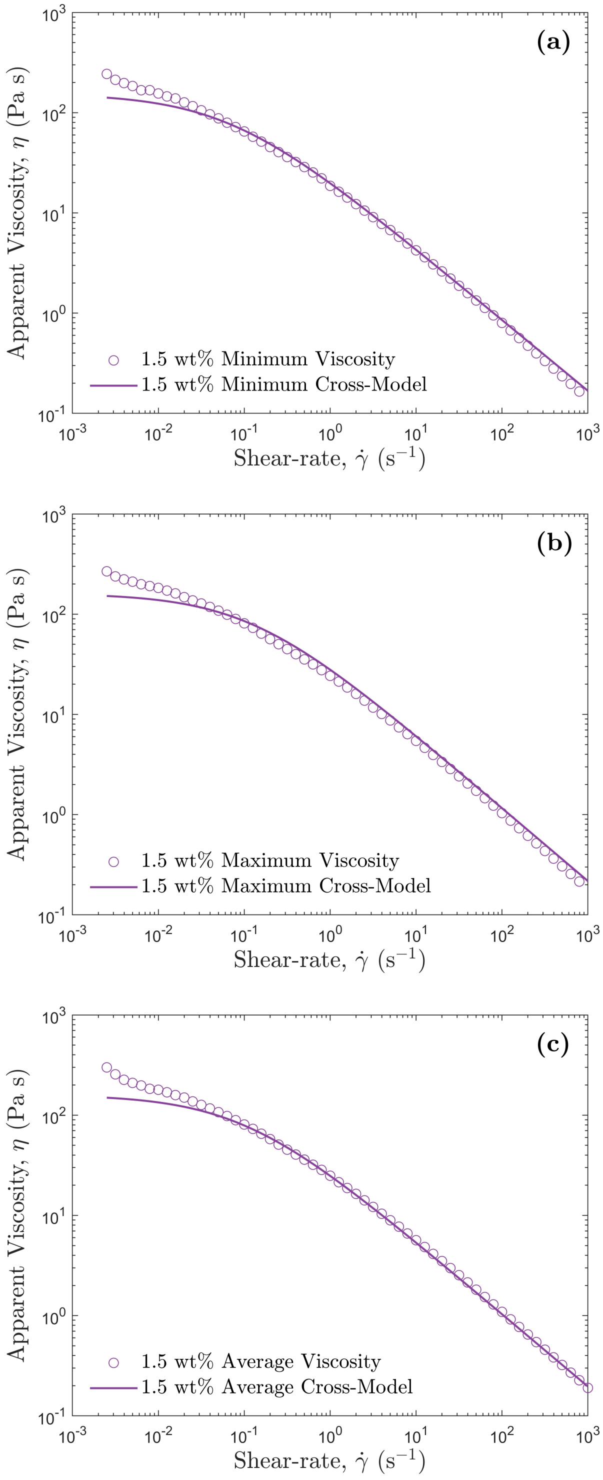

Figure S6. The range in Cross models produced for 1.5 wt% HEC. **(a)** Minimum Cross model. **(b)** Maximum Cross model. **(c)** Average Cross model.

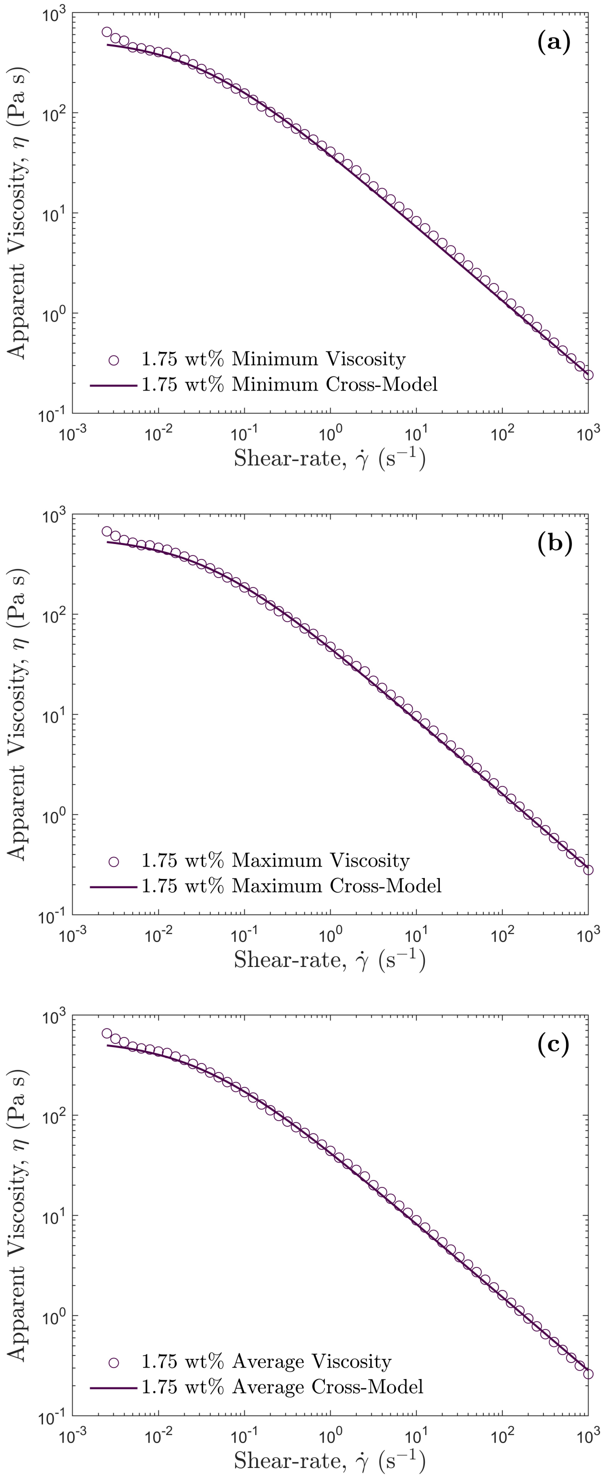

Figure S7. The range in Cross models produced for 1.75 wt% HEC. **(a)** Minimum Cross model. **(b)** Maximum Cross model. **(c)** Average Cross model.

Table S4. Physical properties and Cross model parameters for the three HEC fluids. Cross model parameters defined in Equation 14 include η_∞, η_0, c, and p. Also reported are the density values, range of viscosities observed in the particle settling experiments, and the R-square values for each Cross model fit.

wt %	Cross model	η_∞ (Pa s)	η_0 (Pa s)	c	p	Density, ρ (kg m^{-3})	Range η (Pa s)	R-square
	Average	0.000978	100	16.1	0.660			0.9985
1.25	Minimum	0.000978	69	9.59	0.679	1002.0	2 - 46	0.9996
	Maximum	0.000978	112	18.1	0.639			0.9970
	Average	0.000994	160	10.4	0.725			0.9968
1.5	Minimum	0.000994	155	15.0	0.711	1003.5	9 - 125	0.9967
	Maximum	0.000994	161	8.48	0.731			0.9956
	Average	0.00103	575	32.1	0.734			0.9996
1.75	Minimum	0.00103	555	35.0	0.739	1004.4	38 - 447	0.9993
	Maximum	0.00103	500	29.2	0.742			0.9997

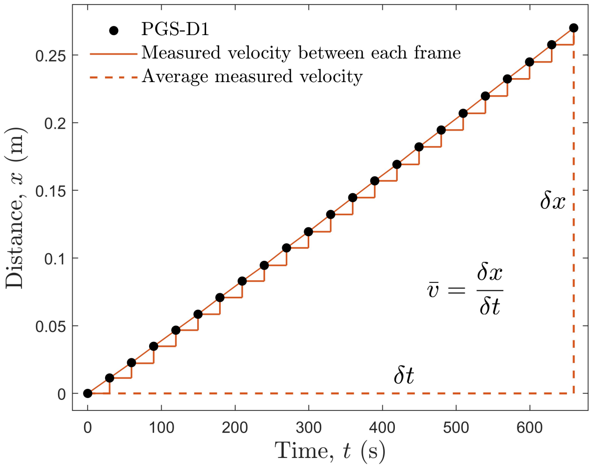

Figure S8. Graphical demonstration of the measured velocity calculations. The solid lines indicate the measured velocity calculated between each frame of the particles settling. This is needed to show any velocity fluctuations in the particle's settling and to ensure that it had reached a terminal velocity. Terminal velocity is demonstrated by a constant velocity gradient, indicating zero acceleration. The dashed line shows how the average settling velocity was calculated.

Table S5. Experimental results for pure golden syrup (PGS) and diluted golden syrup (DGS) particle drops. Reported are the syrup type and experiment number, with the corresponding sphere-to-tube-diameter ratio λ, Reynolds number Re, drag coefficient C_d, measured velocity average v_m, Stokes' velocity v_s, percentage difference between Stokes' and the measured velocity, wall-corrected velocity v_{wc}, and percentage difference between the wall-corrected and the measured velocity.

	Experiment Number	λ	Re	C_d	v_m (m s^{-1})	v_s (m s^{-1})	% difference between v_s and v_m	v_{wc} (m s^{-1})	% difference between v_{wc} and v_m
Pure Golden Syrup (PGS)	PGS-D1	0.0628	0.0000245	977866	0.000409	0.000473	15.5	0.000412	0.59
	PGS-D2	0.0628	0.0000261	918465	0.000419	0.000492	17.5	0.000429	2.35
	PGS-D3	0.0628	0.0000262	916706	0.00418	0.000494	18.2	0.000430	2.92
	PGS-D4	0.0952	0.0000879	273116	0.000906	0.00113	24.3	0.000909	0.27
	PGS-D5	0.0952	0.0000870	275914	0.000900	0.00113	25.3	0.000909	1.02
	PGS-D6	0.0952	0.0000853	281257	0.000883	0.00113	28.2	0.000909	3.38
	PGS-D7	0.158	0.000339	70721	0.00210	0.00313	32.8	0.00215	2.16
	PGS-D8	0.158	0.000336	71438	0.00204	0.00318	35.7	0.00219	6.87
	PGS-D9	0.158	0.000341	70302	0.00209	0.00318	34.7	0.00219	5.17
	PGS-D10	0.206	0.000649	36974	0.00305	0.00535	43.1	0.00321	5.41
	PGS-D11	0.206	0.000642	37408	0.00304	0.00528	42.4	0.00317	4.16
	PGS-D12	0.206	0.000672	35699	0.00317	0.00528	39.9	0.00317	-0.143
	PGS-D13	0.239	0.000963	24933	0.00392	0.00730	46.2	0.00396	0.973
	PGS-D14	0.239	0.000958	25044	0.00391	0.00730	46.5	0.00396	1.42
	PGS-D15	0.239	0.000967	24825	0.00394	0.00730	46.0	0.00396	0.537
	PGS-D16	0.300	0.00157	15330	0.00509	0.0112	55.0	0.00500	-1.31
	PGS-D17	0.300	0.00158	15160	0.00514	0.0112	54.3	0.00500	-2.91
	PGS-D18	0.300	0.00161	14920	0.00523	0.0112	53.5	0.00500	-4.45
Diluted Golden Syrup (DGS)	DGS-D1	0.0628	0.000373	64288	0.00166	0.00179	7.52	0.00156	-6.37
	DGS-D2	0.0628	0.000374	64213	0.00165	0.00179	8.24	0.00156	-5.74
	DGS-D3	0.0628	0.000377	63717	0.00166	0.00180	8.39	0.00157	-5.61
	DGS-D4	0.0952	0.00123	19520	0.00356	0.00405	13.9	0.00327	-8.13
	DGS-D5	0.0952	0.00122	19674	0.00353	0.00405	14.8	0.00327	-7.4
	DGS-D6	0.0952	0.00121	19775	0.00351	0.00405	15.4	0.00327	-6.93
	DGS-D7	0.158	0.00468	5138	0.00818	0.0112	37.4	0.00772	-5.65
	DGS-D8	0.158	0.00470	5107	0.00820	0.0112	37.2	0.00772	-5.80
	DGS-D9	0.158	0.00472	5085	0.00819	0.0113	37.8	0.00775	-5.38
	DGS-D10	0.206	0.00918	2619	0.0122	0.0190	55.5	0.0114	-6.69
	DGS-D11	0.206	0.00917	2622	0.0122	0.0190	55.6	0.0114	-6.61
	DGS-D12	0.206	0.00922	2608	0.0123	0.0190	54.8	0.0114	-7.09
	DGS-D13	0.239	0.0133	1811	0.0152	0.0264	74.0	0.0143	-5.58
	DGS-D14	0.239	0.0136	1776	0.0154	0.0264	71.4	0.0143	-6.98
	DGS-D15	0.239	0.0134	1792	0.0153	0.0264	72.1	0.0143	-6.57
	DGS-D16	0.300	0.0211	1143	0.0192	0.0406	111.8	0.0180	-5.95
	DGS-D17	0.300	0.0212	1134	0.0193	0.0406	110.2	0.0180	-6.66
	DGS-D18	0.300	0.0214	1127	0.0196	0.0406	106.9	0.0180	-8.11

Table S6. Experimental results for all HEC particle drops, using $\dot{\gamma} = v_m/W$. Reported are the HEC concentration and experiment number, with the corresponding sphere-to-tube-diameter ratio λ, Reynolds number Re, drag coefficient C_d, measured velocity average v_m, Stokes' velocity v_s, percentage difference between Stokes' and the measured velocity, wall-corrected velocity v_{wc}, and percentage difference between the wall-corrected and the measured velocity.

	Experiment Number	λ	Re	C_d	v_m (m s^{-1})	v_s (m s^{-1})	Δ between v_s and v_m (%)	v_{wc} (m s^{-1})	Δ between v_{wc} and v_m (%)
1.25 wt%	1.25-D1	0.0628	0.000131	183798	0.00189	0.000827	-56.3	0.000720	-62.0
	1.25-D2	0.0628	0.000129	186758	0.00188	0.000821	-56.2	0.000715	-61.9
	1.25-D3	0.0628	0.000131	183186	0.00190	0.000828	-56.4	0.000721	-62.0
	1.25-D4	0.0952	0.000935	25661	0.00557	0.00298	-46.5	0.00241	-56.8
	1.25-D5	0.0952	0.000900	26668	0.00543	0.00295	-45.7	0.00238	-56.2
	1.25-D6	0.0952	0.000908	26445	0.00546	0.00296	-45.9	0.00238	-56.3
	1.25-D7	0.158	0.0170	1419	0.0258	0.0195	-24.4	0.0134	-48.1
	1.25-D8	0.158	0.0165	1461	0.0254	0.0193	-23.9	0.0133	-47.7
	1.25-D9	0.158	0.0160	1506	0.0249	0.0191	-23.3	0.0131	-47.3
	1.25-D10	0.206	0.0884	276	0.0600	0.0565	-5.90	0.0339	-43.5
	1.25-D11	0.206	0.0904	270	0.0609	0.0570	-6.41	0.0342	-43.8
	1.25-D12	0.206	0.0930	262	0.0620	0.0576	-7.08	0.0346	-44.2
	1.25-D13	0.239	0.284	88.4	0.112	0.117	6.01	0.0636	-42.5
	1.25-D14	0.239	0.304	82.7	0.115	0.120	4.38	0.0654	-43.3
	1.25-D15	0.239	0.300	83.8	0.114	0.120	4.56	0.0650	-43.3
	1.25-D16	0.300	1.76	16.5	0.284	0.348	22.8	0.155	-45.5
	1.25-D17	0.300	1.51	18.8	0.258	0.328	26.9	0.146	-43.6
	1.25-D18	0.300	1.80	16.1	0.288	0.352	22.2	0.156	-45.7
1.5 wt%	1.5-D1	0.0628	0.0000141	1702840	0.000530	0.000318	-40.0	0.000277	-47.8
	1.5-D2	0.0628	0.0000136	1766540	0.000516	0.000316	-38.7	0.000275	-46.6
	1.5-D3	0.0628	0.0000139	1729438	0.000526	0.000317	-39.6	0.000276	-47.4
	1.5-D4	0.0952	0.0000772	311034	0.00149	0.000930	-37.5	0.000750	-49.6
	1.5-D5	0.0952	0.0000781	307150	0.00151	0.000933	-38.0	0.000752	-50.0
	1.5-D6	0.0952	0.0000834	287737	0.00157	0.000945	-39.7	0.000762	-51.3
	1.5-D7	0.158	0.00107	22418	0.00657	0.00484	-26.4	0.00332	-49.4
	1.5-D8	0.158	0.00112	21419	0.00676	0.00492	-27.2	0.00338	-50.0
	1.5-D9	0.158	0.00103	23278	0.00645	0.00476	-26.2	0.00327	-49.3
	1.5-D10	0.206	0.00459	5231	0.0141	0.0126	-10.6	0.00755	-46.4
	1.5-D11	0.206	0.00533	4512	0.0154	0.0133	-13.2	0.00800	-47.9
	1.5-D12	0.206	0.00525	4575	0.0152	0.0133	-13.0	0.00795	-47.8
	1.5-D13	0.239	0.0153	1573	0.0262	0.0266	1.68	0.0144	-44.8
	1.5-D14	0.239	0.0152	1584	0.0263	0.0264	0.519	0.0143	-45.4
	1.5-D15	0.239	0.0133	1804	0.0241	0.0253	4.81	0.0137	-43.1
	1.5-D16	0.300	0.0599	405	0.502	0.0669	33.3	0.0297	-40.8
	1.5-D17	0.300	0.0593	409	0.0498	0.0666	33.6	0.0296	-40.7
	1.5-D18	0.300	0.0654	371	0.0528	0.0693	31.1	0.0308	-41.8
1.75 wt%	1.75-D1	0.0628	0.00000146	16387786	0.000194	0.0000903	-53.4	0.0000786	-59.4
	1.75-D2	0.0628	0.00000148	16163484	0.000196	0.0000905	-53.8	0.0000788	-59.8
	1.75-D3	0.0628	0.00000144	16690149	0.000191	0.0000900	-52.8	0.0000784	-58.9
	1.75-D4	0.0952	0.00000789	3042552	0.000526	0.000266	-49.5	0.000214	-59.2
	1.75-D5	0.0952	0.00000810	2961767	0.000537	0.000268	-50.2	0.000216	-59.8
	1.75-D6	0.0952	0.00000847	2833365	0.000556	0.000271	-51.3	0.000218	-60.7
	1.75-D7	0.158	0.0000982	244421	0.00212	0.00137	-35.2	0.000943	-55.5
	1.75-D8	0.158	0.0000937	256223	0.00206	0.00135	-34.3	0.000929	-54.9
	1.75-D9	0.158	0.0000989	242690	0.00213	0.00138	-35.3	0.000946	-55.6
	1.75-D10	0.206	0.000501	47924	0.00496	0.00396	-22.3	0.00231	-53.4
	1.75-D11	0.206	0.000497	48320	0.00494	0.00385	-22.1	0.00231	-53.3
	1.75-D12	0.206	0.000504	47579	0.00499	0.00387	-22.5	0.00232	-53.5
	1.75-D13	0.239	0.00132	18163	0.00811	0.00742	-8.45	0.00403	-50.3
	1.75-D14	0.239	0.00136	17701	0.00823	0.00749	-8.93	0.00407	-50.6
	1.75-D15	0.239	0.00129	18626	0.00798	0.00735	-7.91	0.00399	-50.0
	1.75-D16	0.300	0.00545	4409	0.0160	0.0190	18.4	0.00843	-47.4
	1.75-D17	0.300	0.00603	3986	0.0170	0.0198	16.1	0.00879	-48.4
	1.75-D18	0.300	0.00586	4100	0.0168	0.0196	16.7	0.00869	-48.2

Table S7. Experimental results for all HEC particle drops, using $\dot{\gamma}_d = v_m/d$. Reported are the HEC concentration and experiment number, with the corresponding sphere-to-tube-diameter ratio λ, Reynolds number Re, Weissenberg number Wi, drag coefficient C_d, measured velocity average v_m, 'new' Stokes' velocity v_{sd}, percentage difference between the 'new' Stokes' and the measured velocity, 'new' wall-corrected velocity v_{wcd}, percentage difference between the 'new' wall corrected and the measured velocity, viscoelastic-corrected velocity v_{ed}, and percentage difference between the viscoelastic-corrected and the measured velocity.

	Experiment Number	λ	Re	Wi	C_d	v_m (m s^{-1})	v_{sd} (m s^{-1})	Δ between v_{sd} and v_m	v_{wcd} (m s^{-1})	Δ between v_{wcd} and v_m	v_{ed} (m s^{-1})	Δ between v_{ed} and v_m
1.25 wt%	1.25-D1	0.0628	0.000329	1.57	72978	0.00189	0.00208	10.05	0.00181	-4.16	0.00188	-0.883
	1.25-D2	0.0628	0.000322	1.55	74522	0.00188	0.002060	9.77	0.00179	-4.41	0.00186	-1.15
	1.25-D3	0.0628	0.000330	1.57	72804	0.00190	0.00208	9.79	0.00182	-4.39	0.00188	-1.11
	1.25-D4	0.0952	0.00215	3.04	11183	0.00557	0.00685	22.9	0.00552	-0.907	0.00594	6.64
	1.25-D5	0.0952	0.00206	2.97	11672	0.00543	0.00674	24.2	0.00544	0.135	0.00585	7.74
	1.25-D6	0.0952	0.00208	2.98	11566	0.00546	0.00677	23.9	0.00546	-0.0786	0.00587	7.51
	1.25-D7	0.158	0.0306	8.50	789	0.0258	0.0352	36.4	0.0242	-6.36	0.0287	11.3
	1.25-D8	0.158	0.0297	8.31	812	0.0254	0.0348	37.3	0.0239	-5.74	0.284	12.0
	1.25-D9	0.158	0.0288	8.09	838	0.0249	0.0344	38.2	0.0236	-5.07	0.0281	12.7
	1.25-D10	0.206	0.133	15.2	184	0.060	0.0851	41.7	0.051	-15.0	0.0665	10.9
	1.25-D11	0.206	0.136	15.4	180	0.0609	0.0858	41.0	0.0515	-15.4	0.0672	10.3
	1.25-D12	0.206	0.140	15.6	175	0.0620	0.0868	40.0	0.0521	-16.0	0.0679	9.59
	1.25-D13	0.239	0.381	24.0	66.7	0.111	0.157	42.3	0.0854	-22.8	0.120	8.51
	1.25-D14	0.239	0.408	25.1	62.4	0.115	0.162	40.1	0.0878	-23.9	0.123	6.98
	1.25-D15	0.239	0.403	24.9	63.1	0.114	0.161	40.6	0.0873	-23.7	0.123	7.31
	1.25-D16	0.3	1.94	49.2	15.1	0.284	0.385	35.7	0.171	-39.7	0.281	-0.947
	1.25-D17	0.3	1.66	44.8	17.3	0.258	0.362	40.2	0.161	-37.7	0.264	2.08
	1.25-D18	0.3	1.99	49.9	14.8	0.288	0.389	35.0	0.173	-40.1	0.284	-1.42
1.5 wt%	1.5-D1	0.0628	0.0000261	0.846	917982	0.000530	0.000590	11.2	0.000513	-3.14	0.000528	-0.335
	1.5-D2	0.0628	0.0000248	0.814	965998	0.000516	0.000562	8.87	0.000489	-5.20	0.000503	-2.48
	1.5-D3	0.0628	0.0000255	0.826	942334	0.000526	0.000566	7.69	0.000493	-6.22	0.000507	-3.53
	1.5-D4	0.0952	0.000146	1.56	164910	0.00149	0.00177	18.9	0.00143	-4.092	0.00152	2.43
	1.5-D5	0.0952	0.000147	1.57	162841	0.00151	0.00178	18.2	0.00144	-4.66	0.00153	1.83
	1.5-D6	0.0952	0.000160	1.65	149982	0.00157	0.00182	16.0	0.00147	-6.45	0.00157	-0.0208
	1.5-D7	0.158	0.00184	4.05	13037	0.00657	0.00833	26.8	0.00572	-12.9	0.00672	2.19
	1.5-D8	0.158	0.00193	4.18	12415	0.00676	0.00850	25.7	0.00584	-13.7	0.00685	1.40
	1.5-D9	0.158	0.00177	3.93	13595	0.00645	0.00818	26.7	0.00561	-13.0	0.00659	2.11
	1.5-D10	0.206	0.00683	6.50	3518	0.0141	0.0187	33.2	0.0112	-20.1	0.0144	2.32
	1.5-D11	0.206	0.00796	7.16	3018	0.0154	0.0199	29.9	0.0120	-22.0	0.0154	0.0322
	1.5-D12	0.206	0.00784	7.09	3062	0.0152	0.0198	30.2	0.0119	-21.9	0.0153	0.232
	1.5-D13	0.239	0.0206	10.8	1168	0.0262	0.0359	37.1	0.0195	-25.6	0.0269	2.64
	1.5-D14	0.239	0.0205	11.0	1176	0.0263	0.0356	35.5	0.0193	-26.4	0.0267	1.48
	1.5-D15	0.239	0.0179	9.69	1342.4	0.0241	0.0340	41.1	0.0185	-23.4	0.0254	5.31
	1.5-D16	0.3	0.0664	16.7	366	0.0502	0.0742	47.9	0.0329	-34.3	0.0525	4.67
	1.5-D17	0.3	0.0657	16.6	369	0.0498	0.0739	48.2	0.0328	-34.2	0.0523	4.88
	1.5-D18	0.3	0.0725	17.6	335	0.0528	0.0768	45.4	0.034	-35.4	0.0545	3.10
1.75 wt%	1.75-D1	0.0628	0.00000282	0.494	8515913	0.000194	0.000174	-10.3	0.000222	14.8	0.000155	-20.0
	1.75-D2	0.0628	0.00000287	0.500	8374335	0.000196	0.000175	-10.9	0.000152	-22.4	0.000156	-20.5
	1.75-D3	0.0628	0.00000276	0.486	8706886	0.000191	0.000173	-9.59	0.000150	-21.3	0.000154	-19.4
	1.75-D4	0.0952	0.0000154	0.884	1559923	0.000526	0.000520	-1.22	0.000419	-20.3	0.000445	-15.5
	1.75-D5	0.0952	0.0000159	0.903	1513193	0.000537	0.000525	-2.19	0.000424	-21.1	0.000500	-16.3
	1.75-D6	0.0952	0.0000167	0.934	1438916	0.000556	0.000535	-3.75	0.000431	-22.4	0.000458	-17.6
	1.75-D7	0.158	0.000171	2.15	140518	0.00212	0.00239	12.8	0.00164	-22.5	0.00190	-10.1
	1.75-D8	0.158	0.000162	2.035	147861	0.00206	0.00235	14.1	0.00161	-21.7	0.00187	-9.20
	1.75-D9	0.158	0.000172	2.14	139502	0.00213	0.00240	12.6	0.00165	-22.7	0.00191	-10.3
	1.75-D10	0.206	0.000755	3.08	31773	0.00496	0.00582	17.2	0.00349	-29.6	0.00440	-11.4
	1.75-D11	0.206	0.000749	3.07	32044	0.00494	0.00580	17.4	0.00348	-29.5	0.00438	-11.3
	1.75-D12	0.206	0.000761	3.10	31544	0.00499	0.00584	17.0	0.00350	-29.8	0.00441	-11.6
	1.75-D13	0.239	0.00179	5.42	13418	0.00811	0.0100	24.0	0.00545	-32.7	0.00739	-8.84
	1.75-D14	0.239	0.00184	5.49	13071	0.00823	0.0102	23.3	0.00551	-33.1	0.00747	-9.26
	1.75-D15	0.239	0.00174	5.34	13765	0.00798	0.00994	24.6	0.00540	-32.4	0.00731	-8.38
	1.75-D16	0.3	0.00605	8.56	3970	0.0160	0.0211	31.5	0.00937	-41.6	0.0146	-8.86
	1.75-D17	0.3	0.00670	9.09	3588	0.0170	0.0220	29.0	0.00976	-42.7	0.0153	-10.4
	1.75-D18	0.3	0.00651	8.94	3691	0.0168	0.0217	29.7	0.00965	-42.4	0.0151	-9.97

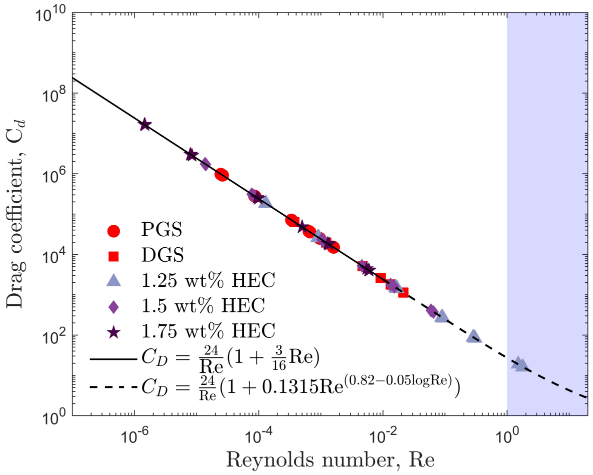

Figure S9. The Re-Cd range of all experiments in this study. The lines denote model relationships based on Equation 4 (solid) and Equation 5 (dashed), for the respective Re values. The purple shaded area shows the higher Reynolds number region where Re > 1, and Equation 3 is no longer valid. Only experiments 1.25-16, 1.25-D17, and 1.25-D18 fall into this range. The rest of the experiments performed as part of this study fall in the Reynolds number range of Re < 1 where Equation 3 is valid.

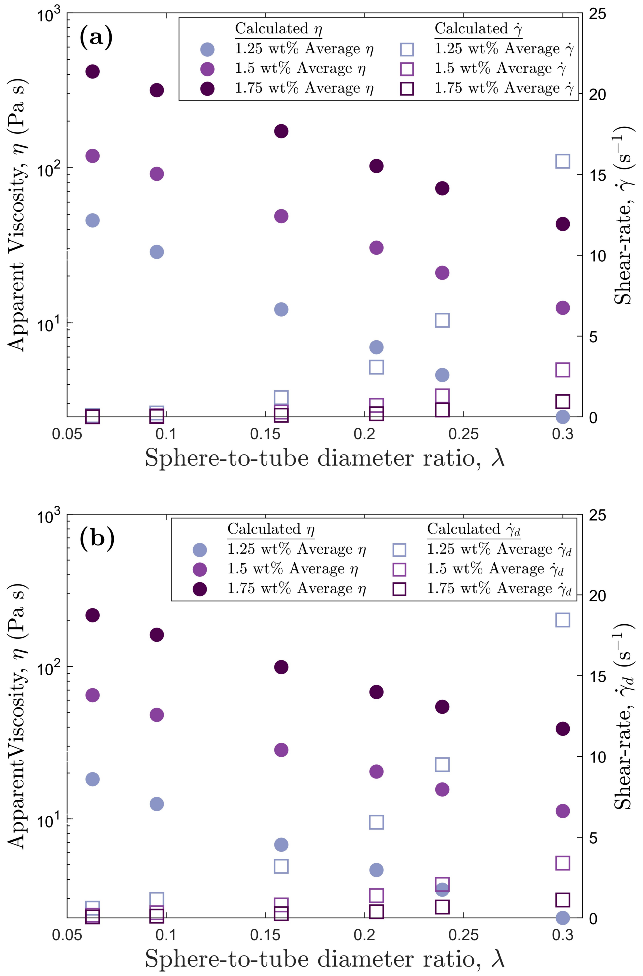

Figure S10. The relationship between the sphere-to-tube diameter ratio, λ, shear-rates, $\dot{\gamma}$ and $\dot{\gamma}_d$ (i.e., Equations 13 and 18 respectively), and apparent viscosity, η. The squares represent the shear-rate values, and the circles represent the viscosity values. **(a)** Viscosities calculated from $\dot{\gamma}$. **(b)** Viscosities calculated from $\dot{\gamma}_d$.
